# State of the Art of Underwater Active Optical 3D Scanners

**DOI:** 10.3390/s19235161

**Published:** 2019-11-25

**Authors:** Miguel Castillón, Albert Palomer, Josep Forest, Pere Ridao

**Affiliations:** Computer Vision and Robotics Research Institute (VICOROB), University of Girona, 17003 Girona, Spain

**Keywords:** underwater 3D laser scanners, 3D reconstruction, active 3D techniques, underwater imaging, underwater robotics

## Abstract

Underwater inspection, maintenance and repair (IMR) operations are being increasingly robotized in order to reduce safety issues and costs. These robotic systems rely on vision sensors to perform fundamental tasks, such as navigation and object recognition and manipulation. Especially, active optical 3D scanners are commonly used due to the domain-specific challenges of underwater imaging. This paper presents an exhaustive survey on the state of the art of optical 3D underwater scanners. A literature review on light projection and light-sensing technologies is presented. Moreover, quantitative performance comparisons of underwater 3D scanners present in the literature and commercial products are carried out.

## 1. Introduction

Oceans cover 71% of the Earth’s surface, of which 95% is still unexplored [1]. Oceanic exploration is a growing field but diving deeper than 50 m poses a huge challenge to human divers. Even though divers can use human-operated vehicles (i.e., submarines) to go deeper, there are still safety issues involved. On the other hand, robotic systems called unmanned underwater vehicles (UUVs) have been experimentally tested at full ocean depth (around 11,000 m) [2]. Their main advantage is that they can perform missions in a safer way and at a lower cost [3].

UUVs can be classified into remotely operated vehicles (ROVs) and autonomous underwater vehicles (AUVs). ROVs are connected to a vessel through a tether that transmits power and control signals for navigation or other tasks. AUVs do not need any pilot nor tether, so their operation vessels can be smaller and less costly. Their diving time is only restricted by the autonomy of the on-board batteries and the required power, so their missions can usually last for several hours [4]. There is a great abundance of marine applications currently carried out by robots, such as underwater archaeology [5,6], ocean monitoring [7,8], marine biology [9] and geology [10,11], damage assessment [12,13] and inspection, maintenance and repair (IMR) applications [14,15], to name a few.

Underwater robots require a large number of modules, each of them aimed at fulfilling a different requirement: IMUs for navigation, motors for thrust, acoustical or optical modems for communication, among others. Apart from all these, a key ability of an autonomous robotic system is sensing its environment. For UUVs it is especially important to acquire 3D data of its surroundings in order to perform tasks such as object recognition [16], inspection [17], manipulation [18] or navigation [19].

Most of the 3D sensing systems in the literature are either based on acoustic (sonar) or light signals (lidar). Sonars can work at a much longer range (of up to some thousands of meters) and they are not affected by water turbidity. On the other hand, optical sensors provide a much higher lateral resolution and refresh rate [20]. Their short-range (typically a few meters) does not limit the UUV’s performance for intervention tasks since the robot needs to get close to the target.

Optical 3D sensors can be categorized as active or passive. According to Bruno et al. [21], an underwater sensor is said to use active (or structured) light when it projects light patterns onto the scene in a controlled way. These patterns can be a point, a line or more complex shapes. In active techniques, the information given by the structure of the pattern is key to reconstruct the scene in 3D. It is worth noticing that the pattern’s structure is not limited to the spatial domain, but it can also be temporal (which is the base for time of flight (ToF) sensors). Active techniques determine the 3D position of the points in the environment either by ToF or by triangulation principles (see Section 3). On the other hand, passive lighting relies solely in ambient light to illuminate the scene, although artificial diffuse light may be used in dark environments [21] (This definition of active and passive lighting is characteristic of underwater sensors [21,22,23]. For in-air sensors, any projection of artificial light onto the scene is considered an active technique [24]). Passive techniques in underwater environments typically use stereo vision or structure from motion (SfM).

**Figure 1:**
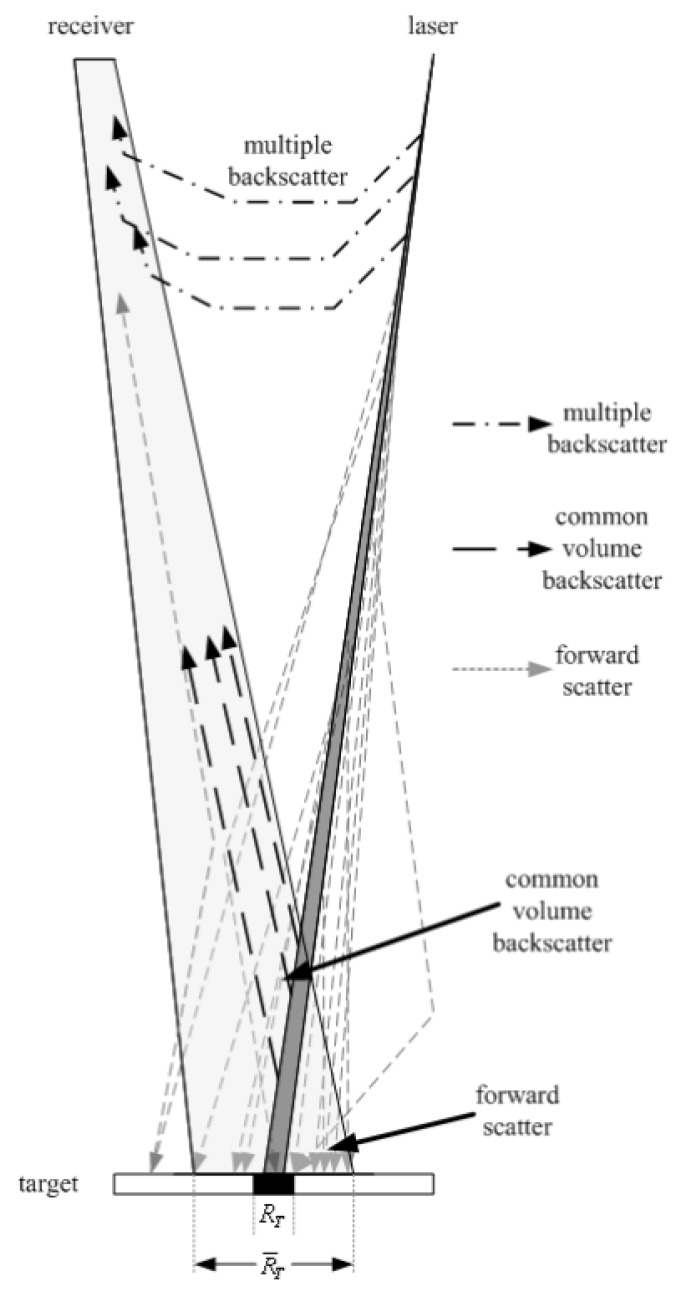
Possible trajectories that laser light can follow while scanning a target [28]. Forwardscatter reduces the lateral resolution. Backscatter leads time of flight (ToF) sensors to range errors.

The main advantages of passive sensors such as passive stereo systems [22,25] and photometric stereo [26,27] are their low price and their theoretical high lateral resolution, which is mainly limited by forward-scattered light [28] (see Figure 1). Their main drawbacks are that they are computationally demanding and rely heavily on the target’s texture to extract features from the image. Nevertheless, they are widely used by the marine research community [6,29,30,31,32]. On the other hand, active 3D sensors typically achieve a much higher point-cloud density, especially in low-contrast scenarios [5]. One of their main drawbacks is that their performance decreases under bright sunlight. Nonetheless, since sunlight is quickly attenuated by ocean water, this is usually not a major problem in relatively deep underwater environments.

Several surveys on underwater imaging systems have been published in the last two decades. In 2001, Jaffe et al. [33] summarized the history of underwater optical imaging and its relationship to other fields of ocean optics, focusing on technological advances in the last decade of the twentieth century. Kocak and Caimi [34] reviewed the historical progress of underwater imaging, with a special focus on the period from 2000 to 2005. Caimi et al. [35] made a survey on underwater optics in 2008, where they covered the advances in image formation and image processing methods, extended range imaging techniques, spatial coherency, and multi-dimensional imaging. Bianco et al. [22] compared the performance of two 3D underwater sensors (based on structured light and passive stereo, respectively) in 2013. In 2015, Massot-Campos and Oliver-Codina [20] presented a very complete review on underwater optical 3D reconstruction, including a quantitative comparison of performance criteria. The present survey reviews the state of the art of active underwater 3D optical sensors, focusing especially on the technologies for light projection and light sensing. Their working principles, as well as their practical limitations, are explained. Moreover, quantitative performance comparisons of underwater 3D scanners present in the literature and of commercial products are carried out.

This paper is structured as follows: the main challenges that underwater 3D sensors have to face are summarized in Section 2. Methods to reconstruct 3D scenes are explained in Section 3. Current technologies used for projecting light are gathered in Section 4. Next, the existing underwater 3D active optical sensors that can be found in the literature after 2015 are compared quantitatively in Section 5. Finally, the conclusions drawn by the authors concerning subsea 3D imaging are collected in Section 6.

## 2. Challenges of Underwater Imaging

**Figure 2:**
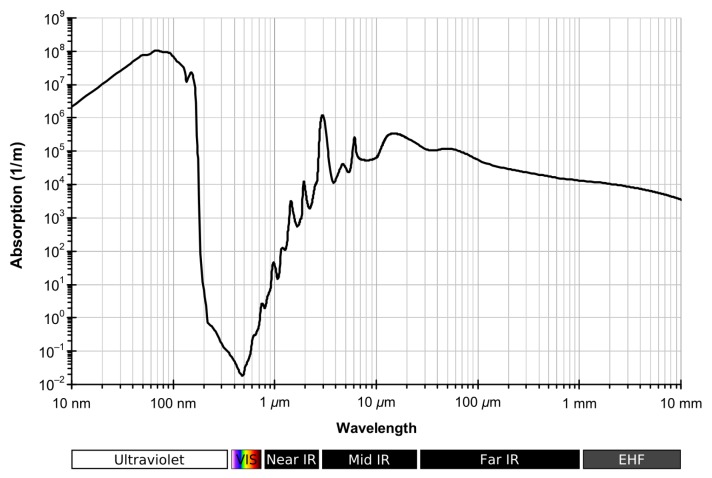
Water absorption spectrum of light [40].

One of the main challenges of underwater imaging is that light is strongly attenuated by water. This process is wavelength-dependent (see Figure 2). The visible spectrum can travel up to some hundreds of meters before being completely absorbed by water. infrared (IR) wavelengths, on the other hand, do not propagate further than 30 cm [36,37]. Images taken in shallow waters (with a depth of less than 10 m) are less affected by water attenuation. However, there are other phenomena that degrade those images, such as flickering [4,38] and higher backscatter due to the presence of suspended particles: just like fog does above water, the floating particles and organisms randomly distributed in the water reflect the projected light back to the sensor and dazzle it [39] (see Figure 1).

Furthermore, vision systems are usually enclosed inside a sealed casing with a transparent viewport. This entails that light suffers a refraction process twice before arriving at the camera from the scene, according to Snell’s law [41]. This complicates further the computation of the 3D position of the observed object (see Section 2.1).

Two concepts are commonly used to characterize the underwater environment when testing an underwater sensor: turbidity and attenuation length. Turbidity is the cloudiness of a fluid caused by its suspended particles, and it is measured in ntu [42]. The attenuation length of a beam of particles (in this case, light) is defined as the distance where the intensity of the beam has dropped to 1/e (≈37%) of its initial intensity [43].

**Figure 3:**
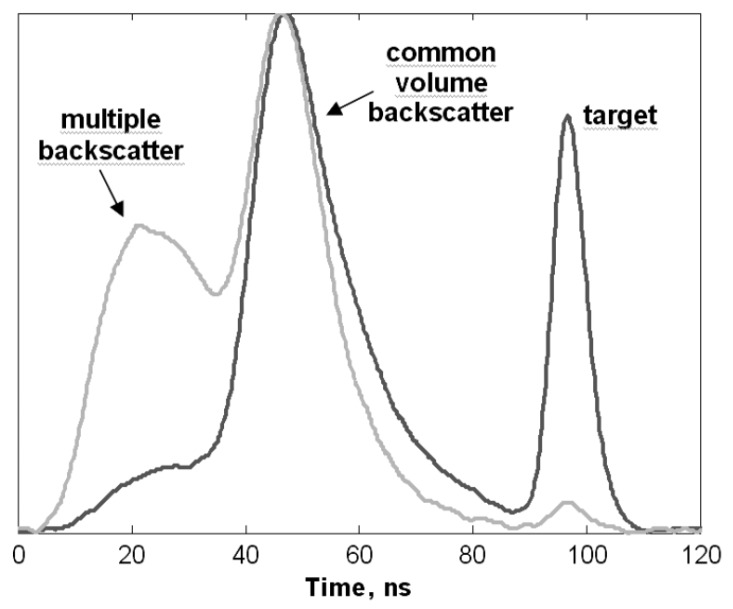
Typical measurement of a ToF sensor in a scattering medium, like water, for two different turbidity levels [28]. The vertical axis is the magnitude of the output signal of the ToF sensor, which relates to the intensity of the received light at each time instant. Higher turbidity (lighter curve) implies a higher peak from multiple backscatter and a very low target peak, which affects the accuracy of the measurement. If a range-gated sensor is used, its optimal opening time to accurately detect the target is around 90 ns.

All these optical differences between air and water entail that 3D sensing technology developed for in-air applications cannot be directly submerged and used for underwater tasks. The design of underwater scanners usually includes some of the following approaches to tackle the medium-specific challenges:

- The amount of light scattered back from suspended particles to the vision system can be reduced by increasing the baseline, which is the separation distance between the light source and the sensor. However, there is a limit to this increment defined by the maximum sensor size that the AUV can carry [44].
- A range-gated receiver synchronized with the laser system can also help differentiate between the backscattered noise and the light reflected by the target [45] (see Figure 3).
- Acquiring a pair of images using a polarizer at different orientations enhances the image contrast [46].
- Light wavelengths with low absorption under water can propagate longer distances. These wavelengths correspond to green or blue, but green laser sources are usually preferred because they are cheaper and more energy-efficient [47].
- Lasers sources permit a more efficient propagation when compared to diffuse light because they are highly collimated and have a high optical density [48].

### 2.1. Calibration

Calibration is a fundamental step in any vision system aimed at acquiring undistorted, accurate and reliable data and it usually comprises two steps. First, the intrinsic parameters of the camera (including the lens) must be computed [49]. Second, the position and orientation (extrinsic parameters) of the camera with respect to the laser projector (in case of a laser triangulation system) or with respect to the other camera (in case of stereo vision) must also be determined.

Underwater camera calibration has been widely studied in the literature. For instance, Shortis [50] presented a very complete survey on calibration techniques and considerations for underwater photogrammetric systems, and Sedlazeck and Koch [51] compared perspective and non-perspective camera models in underwater imaging. The calibration parameters of a vision system change depending on external conditions: depth, temperature, and salinity change the refractive index of water [52,53,54]. On top of that, the shape of the camera housing is prone to deformations at increasing pressure levels [30].

**Figure 4:**
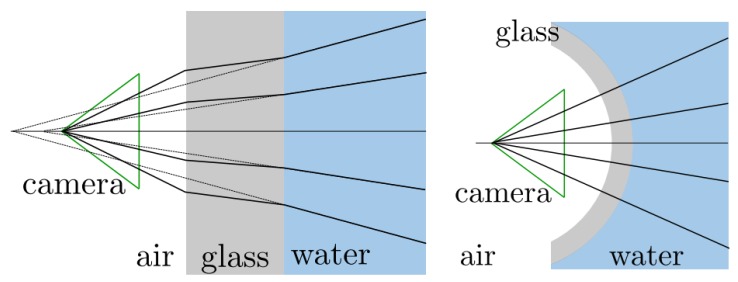
Refraction of light rays at flat and dome ports, respectively [51].

Refraction provokes a pin-cushion distortion, which makes that the largest reconstruction errors appear at the edges of the target [55]. Due to the symmetric nature of this effect, it can be absorbed by the radial lens distortion component of the calibration parameters [50]. A practical method for calibrating a camera for underwater laser scanning is presented in [56]. However, the refraction effect entails systematic errors, since the assumption of a single projection centre for the camera (single view-point (SVP)camera model) does not hold (see Figure 4) [51]. A more complicated approach that can be followed in order to solve this issue is tracing the light rays through the refractive interfaces, such as in [57].

Underwater cameras mainly use two types of ports: flat or dome-shaped. At the expense of a more costly and difficult process of manufacture and assembly, dome ports can in principle reduce the refractive effect because there is a theoretical alignment between the interface normal and the incoming rays. However, due to small misalignments, this reduction is not usually total [51]. Performance comparisons of camera models and types of ports are done in [58,59]. Similarly, projected light also suffers this refraction process. For instance, Palomer et al. [57] demonstrated that an elliptic cone is a better geometry to describe the deformation of a laser line through a flat port in water than a plane, especially when the incidence angle between the laser and the port increases. Using an elliptic cone rather than a plane, however, makes the 3D reconstruction process more computationally demanding.

### 2.2. Open Issues

To the present date, underwater active optical 3D scanners in the literature lack two important abilities for UUVs’ tasks:

- First, the data refresh rate of these sensors is too low for real-time applications in which highly dense point clouds are required. Acquisition time is important because it limits the accuracy of the 3D sensor. The relative motion during that period entails reconstruction errors. Consequently, a longer time means a larger error. One solution to mitigate this problem consists of using a very accurate and fast-refreshing navigation system, such as an inertial navigation system (INS). However, these devices have the disadvantage of being very expensive. Another approach is allowing an increase of the scanner’s frequency by reducing either its field of view (FoV) or its lateral resolution. Other sensors use one-shot reconstruction so that the whole scene is captured at once, but backscatter effects and processing limitations bound the maximum lateral resolution [60]. While these approaches may be valid for certain conditions, a faster refresh rate is key to enable scanners to be mounted on realistic moving platforms.
- Second, these devices are generally not able to sense the color of the surrounding objects. Obtaining characteristics of the environment aside from its geometric description, such as the texture of each point, can be relevant in applications dealing with autonomous manipulation. Bodenmann et al. [61] developed a laser system that enables the simultaneous capture of both structure and color from the images of a single camera and tested it for a mapping application. Nonetheless, it does not seem directly suitable for autonomous object manipulation, since the position of the laser plane with respect to the camera is fixed. Performing laser beam steering would reduce the scanning time significantly. Another existing method was presented by Yang et al. [62]. They used three lasers (RGB) to retrieve both color and 3D position of the point cloud. However, it cannot produce accurate color information as it returns three thin spectral peaks of light as opposed to a broad spectrum. As commercial products, Kraken Robotics [63] claims to have developed a working system similar to [62], which can be mounted on an UUV. It is important to note that, in general, the perceived color of an underwater scene or object is not the same as outside the water since the water absorption index of light depends heavily on its wavelength. Therefore, a color restoration process is usually needed [64,65,66,67].

There are other aspects that can be potentially improved, such as laser peak detection [68]. Several approaches have been proposed that use filters to deal with undesired lighting peaks, which are typical in underwater imaging [69,70]. Further refining the accuracy of the laser peak detection means improving the accuracy of the 3D reconstruction, which is especially relevant in media with high noise level like turbid water.

## 3. 3D Reconstruction Methods for Active Optical Sensors

Most of the devices at the receiving end of underwater active optical 3D sensors in the literature are based either on ToF or on triangulation methods. The general principles of both approaches are explained in Section 3.1 and Section 3.2, respectively. In Section 3.3, their main characteristics are compared and the challenges of their underwater implementations are described.

### 3.1. Time of Flight

ToF sensors compute the depth *d* of a point by measuring the time Δt from the emission of a light ray until its reception, according to:

(1) $$d=\frac{1}{2}\;\Delta t\;c_{m}.$$

The factor of ½ is due to the fact that light travels a distance 2d until it arrives back to the sensor. Here, $c_{m}$ is the speed of light in the medium where the sensor is immersed and it is given by the absolute refraction index of the medium $\eta_{m}$ and the speed of light in vacuum $c\approx 2.9979\times 10^{8}$ m/s [41]:

(2) $$c_{m}=\frac{c}{\eta_{m}}.$$

Apart from positioning a point in the depth direction, its position in the other two axes needs to be determined in order to locate it in the 3D space. That spatial information can be retrieved using three different configurations:

- A punctual light source steered in 2D, along with a single detector.
- A linear light source swept in 1D, along with a 1D array of detectors.
- Diffuse light that illuminates the whole scene at once, along with a 2D array of detectors.

**Figure 5:**
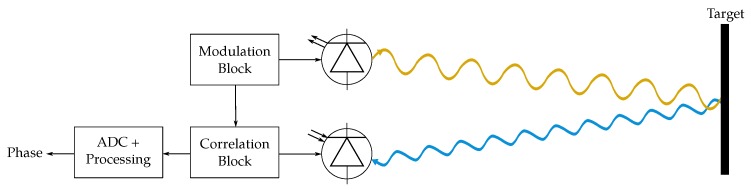
Scheme of a ToF 3D sensor using diffuse illumination and a 2D receiver.

Most of the ToF sensors in the literature share a similar basic structure [71] (see Figure 5): a light source (transmitter), a matching detector (receiver), suitable circuitry to provide the needed power supply and control signals to transmitter and receiver, readout electronics and an analog–digital converter (ADC).

The elapsed time Δt can be measured directly. However, due to the high speed of light, very accurate timers are needed: for a depth resolution of 1 mm, the accuracy of the time measurement mechanism needs to be better than 7 ps. There are some alternatives to try to obtain more accurate measurements:

- One of them is using a continuous wave (CW)-modulated light, so that the phase difference between the sent and received signals can be measured. As the modulation frequency is known, this measured phase difference corresponds to the time of flight [72].
- Another approach consists in using pulsed light. Pulsed light has a high signal to noise ratio, which makes the system more robust to background illumination. light emitting diodes (LEDs) and laser diodes are commonly used to generate pulses with repetition rates on the order of tens of kHz.

The reader is referred to [71,73,74] for more detailed explanations on ToF sensors, including data processing and calibration.

#### Receiver-End Technologies

Different technologies are used at the receiving end of ToF sensors. Range-gated cameras have the advantage of being more robust to backscatter effects [75]. More specifically, gated intensifiers coupled to charge-coupled device (CCD) image sensors allow for precise range gating. However, complementary metal-oxide-semiconductor (CMOS) chips achieve high frame rates and high spatial and depth resolution with a lower system complexity [76].

Apart from cameras, there are other sensing ToF technologies. Four of them are now briefly reviewed, namely PIN photodiodes, photomultiplier tubes (PMTs), avalanche photodiodes (APDs) and silicon photomultipliers (SiPMs).

- A PIN photodiode is a diode with an intrinsic semiconductor in the middle of a PN union that is sensitive to the incidence of light [77]. Its usage is rather limited due to its unity gain: only one electron is generated for each detected photon, which bounds its signal-to-noise ratio (SNR). Since conventional PIN photodiodes are much easier and cheaper to fabricate than other technologies and highly reliable all the time [78], they are used in very price-sensitive applications where gain is not a critical factor, such as timers in pulsated lidar [79]. Its bandwidth is up to 10 GHz [78].
- APDs can detect smaller quantities of light than PIN photodiodes, since their gain is around 10–100, which also improves their SNR. Their bandwidth is also high, of around 40 GHz [78]. They have been rarely used for underwater 3D measurements [80].
- PMTs present much larger gains of around $10^{6}$–$10^{8}$, while maintaining a high bandwidth. Their main disadvantage is their fragility and extremely high sensitivity to ambient light, which can damage the device irreversibly. They have been used in underwater robotics [81,82].
- SiPMs are composed of multiple single-photon avalanche photodiodes (SPADs), which are APDs in Geiger mode aimed at detecting single photons [83]. They are commercialized by Hamamatsu under the name multipixel photon counter (MPPC) [84]. They have a large gain of around $10^{6}$, although their bandwidth is lower [85]. Despite being used for in-air LiDAR sensors [86], they have not been mounted on underwater 3D scanners.

### 3.2. Triangulation

Active light triangulation sensors find the 3D position of a point in the scene by combining geometrical information from the light emitter and the receiver. This way, the position in space of the scanned point coincides with the intersection of two light rays: the one sent by the projector and the one going from the camera focal point through the illuminated pixel, assuming the camera has been simplified by a pin-hole camera model. Since all the needed information is known, finding the 3D position of a point comes down to solving a geometry problem. The derivations for a point laser scanner and a line laser scanner are done in Section 3.2.1 and Section 3.2.2, respectively.

Regarding the used technologies, most underwater triangulation-based scanners use regular cameras protected inside a sealed housing. The difficulties that this fact entails have been explained in Section 2.1. Nevertheless, a new type of sensors called event cameras are being gradually used in in-air 3D robotic vision systems for scene reconstruction and tracking [87,88]. Instead of storing a full image frame at defined intervals, event cameras record an asynchronous sequence of per-pixel intensity changes, each with a precise time stamp [89]. Their low power consumption, high frame rate and absence of motion blur [90] postulate them as appropriate sensors for very agile robots [91].

#### 3.2.1. Point Triangulation Scanners

**Figure 6:**
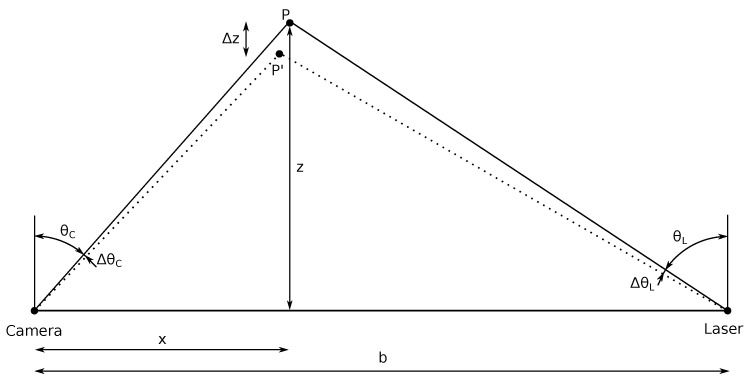
Scheme of the geometrical relationships used to compute the distance by point triangulation scanners.

The principle of calculating the depth of a point is based on the simplified 2D triangulation scheme depicted in Figure 6, where the following relationships hold for an arbitrary point *P*:

(3) $$\tan\theta_{C}=\frac{x}{z}$$

(4) $$\tan\theta_{L}=\frac{b-x}{z}$$

Substituting *x* from the first equation into the second one and rearranging:

(5) $$z=\frac{b}{\tan\theta_{L}+\tan\theta_{C}}.$$

Therefore, calculating the *z* coordinate of a point requires knowing the baseline of the sensor, the angle at which the laser beam is steered and the angle that the reflected light ray makes with respect to the camera (which is given by the pixel position).

Designers of triangulation systems have to decide the geometrical configuration of its elements (regardless of them being a camera and a laser, a camera and a projector or two cameras) to comply with the requirements of depth resolution and maximum scanning range. In order to explain the concept of depth resolution geometrically, consider now that both the camera and the laser in Figure 6 are non-ideal and have finite angular resolution ($\Delta \theta_{C}$ and $\Delta \theta_{L}$, respectively). This entails that the observable FoV is discretized and measurements can only happen in the limited intersections of light rays coming out of the laser and sensed by the camera. Of all those intersections, the closest to *P* is $P^{\prime }$. Their *z* coordinates differ by Δz, which is the depth resolution of the device at point *P*. The relationship of depth resolution with scan distance, baseline, and angular resolutions is given by the following equation [92]:

(6) $$\Delta z=z-\frac{b}{\tan\left(\theta_{L}+\Delta \theta_{L}\right)+\tan\left(\theta_{C}+\Delta \theta_{C}\right)}$$

**Figure 7:**
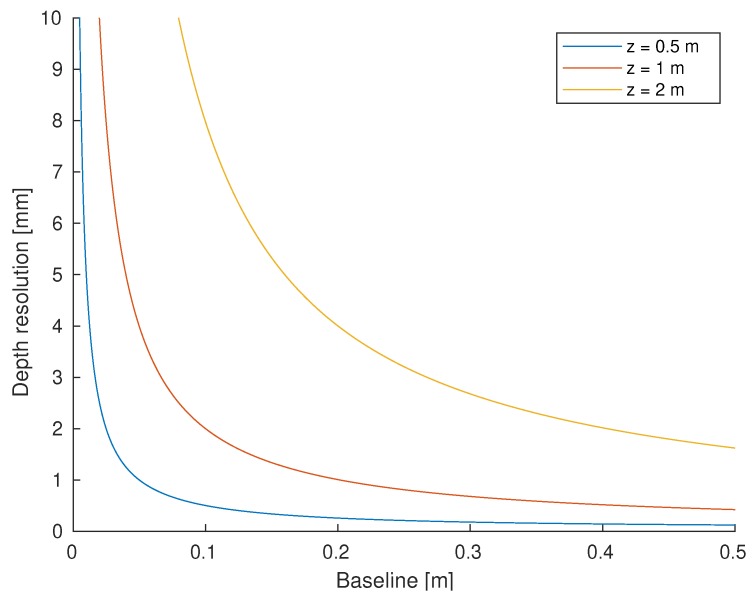
Depth resolution versus baseline for different scan distances.

In order to express this relationship graphically, the *x* coordinate of point *P* is assumed to be halfway between the camera and the laser, which means that x=b/2 and $\theta_{L}=\theta_{C}$. Moreover, the values of the angular relationships are assumed to be $\Delta \theta_{L}=\Delta \theta_{C}=0.1$ mrad. The resulting plot is depicted in Figure 7. The main conclusion is that both scanning at larger distances and using a shorter baseline influence negatively in the depth resolution.

Therefore, considering the mechanical design of the sensor, an approach to obtain measurements with finer depth resolution consists of increasing the baseline, which will especially benefit longer distance scans. However, there are two main limitations to this increment: the maximum size of the sensor that can be mounted on an UUV and the fact that a larger baseline entails a higher probability of occlusions in the short-range [93].

#### 3.2.2. Line Triangulation Scanners

The principle of laser line scanners (LLSs) is similar to point scanners, but they project a plane onto the scene. The intersection of this plane with an object creates an illuminated line that is registered by the camera. The 3D position in space of each line with respect to the camera coordinate frame can be calculated by combining information of the laser plane with the camera pixels [20,94,95].

**Figure 8:**
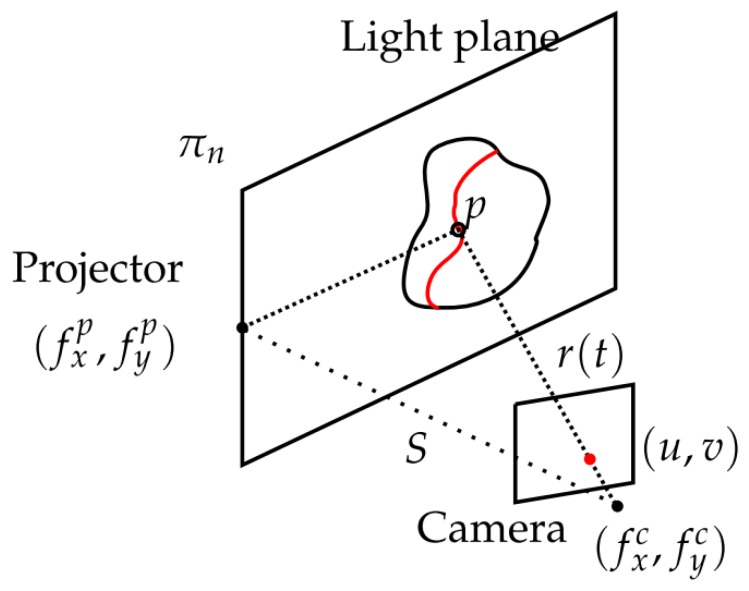
Line triangulation [20].

The equation of the laser plane is assumed to be known with respect to the camera coordinate frame and can be written as π≡Ax+By+Cz+D=0. The parametric equation of any point *p* of the line (see Figure 8) in the camera coordinate frame is

(7) $$r\left(t\right)=\left[\begin{array}{c} \frac{u-c_{x}}{f_{x}}t\\ \frac{v-c_{y}}{f_{y}}t\\ t \end{array}\right],$$

where $\left(f_{x},f_{y}\right)$ is the camera focal length in the *x* and *y* axes, $\left(c_{x},c_{y}\right)$ is the position of the central pixel in the image and (u,v) is the position of one of the detected pixels in the image.

In order to find the intersection of π with r(t), both equations are combined. Noting that z=t, the depth of *p* from the camera is:

(8) $$z=\frac{-D}{A\frac{u-c_{x}}{f_{x}}+B\frac{v-c_{y}}{f_{y}}+C}.$$

The rest of the 3D components of *p* are directly obtained from Equation (7).

### 3.3. Conclusions

The main difference in performance between ToF and triangulation sensors concerns scan range and depth resolution. The depth resolution of a ToF scanner depends on the resolution of the time or phase measurement but not on the scan distance, unlike for triangulation scanners. This fact was studied by McLeod et al. [96], who identified a threshold of ranges (<1 m) where triangulation-based sensors provided higher depth accuracy. Above 2.5 m, ToF sensors were generally more accurate.

**Figure 9:**
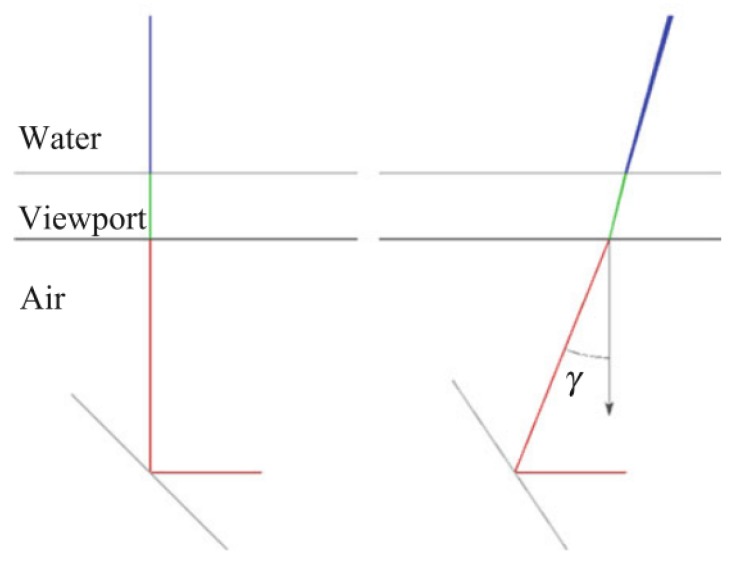
Change in direction of a laser line entering water due to double refraction [57].

**Figure 10:**
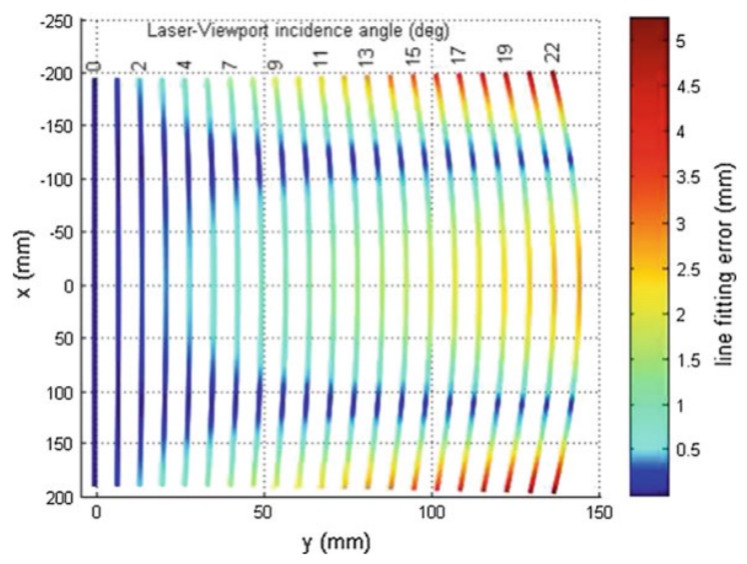
Projected laser lines are increasingly distorted for higher incidence angles of the laser in the viewport [57].

Moreover, implementing any of these measuring methods in an underwater sensor entails a number of challenges. As explained in Section 2.1, the main difficulty to accurately reconstruct the 3D position of a point has to do with the fact that every light ray travels through three different media, namely air, the viewport material, and water. This affects each method in a different way:

- In underwater triangulation sensors using flat viewports, the direction of light rays changes twice due to double refraction (see Figure 9), which can affect the accuracy of the reconstruction. At increasing incidence angles of the laser in the viewport, the laser plane transforms into an elliptic cone (see Figure 10), which makes the 3D reconstruction more computationally demanding [57].
- Along with the previous effect, underwater ToF sensors also suffer from the fact that the different propagation speeds of light (Equation (2)) affect the computation of the total travelled distance (Equation (1)). A detailed implementation of these geometrical calculations can be found in [97].

## 4. Active Light Projection Technologies

This section describes the different types of technologies for active light projection. Active optical 3D scanners project light in a known direction and/or at a known instant in time, which gives essential information for the 3D scene reconstruction. Their main advantage in underwater applications is that they help provide a more homogeneous, denser point cloud, less dependent on the scene’s texture than passive methods such as stereo vision.

**Figure 11:**
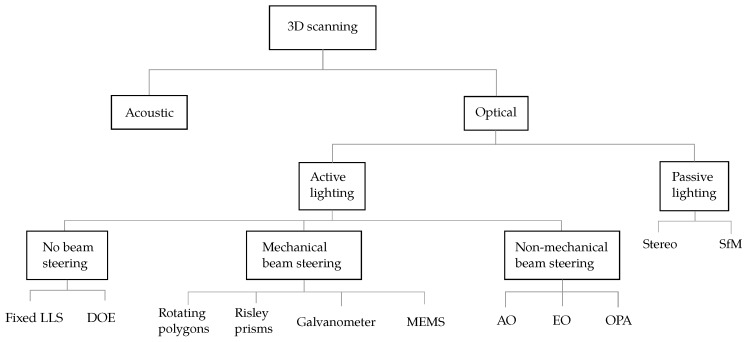
Classification of 3D scanning techniques.

A big-picture classification of beam steering technologies is presented in Figure 11. First, active techniques that do not perform beam steering are explained in Section 4.1. Then, laser steering technologies are classified according to whether they involve mechanical elements or not. Both groups are explained in Section 4.2 and Section 4.3, respectively. Finally, a comparison is carried out in Section 4.4.

Scanning technologies can also be classified in raster and random-access scanning. Raster scanners (such as polygon mirrors or MEMS in resonant mode) need to steer the flying spot or line through the entire FoV before they can start to scan again. On the other hand, random-access scanners can dynamically modify the scanned FoV in order to increase spatial resolution or decrease acquisition time, which makes them more flexible.

Usually, the main performance criteria of laser scanners are scanning speed, fov, resolution, and accuracy. There are other important factors, such as optical efficiency or size.

### 4.1. No Beam Steering

**Figure 12:**
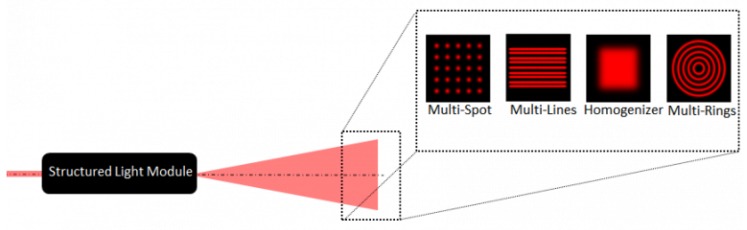
Different types of diffractive optical element (DOEs) [98].

This section gathers two light projection techniques that do not use any type of beam-steering mechanism. On the one hand, the whole scene can be illuminated at once using a homogeneizer diffractive optical element (DOE) (see Figure 12). This technique needs a 2D ToF sensor in order to resolve the 3D position of the scanned scene [75,76]. This is considered an active method because, even though the light direction is not actively controlled, its time structure provides information for the 3D reconstruction. A variation was introduced by Massot-Campos and Oliver-Codina [60], who used a special doe to project a pattern of lines and resolved their 3D position by triangulation. Acquiring the whole scene at once has the advantage of being robust against the high dynamics of the scanned scene and against the sensor movement. However, it usually comes at the cost of reducing lateral resolution.

On the other hand, a fixed laser line scanner (also called profiler) can be used. This setup usually consists of a laser line module and a camera. The relative position of the projected laser plane with respect to the camera is always the same. Even though the laser light is not swept across the scene, it is considered an active technique because the plane equation of the light is essential to reconstruct the 3D points. This 3D reconstruction is usually done by triangulation. This configuration makes these scanners simpler to build and calibrate, but they always need to be attached to a moving platform, usually either an UUV or a rotating tripod. Therefore, the accuracy of the final reconstruction depends greatly on the accuracy of the pose of the platform.

### 4.2. Mechanical Beam Steering

Mechanical beam steering is achieved by moving an object, usually a mirror, in a controlled way. A laser beam can also be steered by moving the whole sensor (for instance, a hand-held scanner). However, the smaller the inertia of the moving part, the faster the scanning can take place. Also, if the moving element is surrounded by air, the friction forces are smaller than in water. Hence, the moving part of an underwater scanner is usually placed inside a sealed housing. In all mirror systems, the reflection angle is twice the mirror tilting angle. Consequently, mechanical scanners can achieve high deflection angles more easily. Moreover, mechanical systems are usually suitable for a wider range of laser wavelengths than solid-state beam steerers, since the mirror’s surface generally has very broadband reflectance.

#### 4.2.1. Rotating Polygon Mirror

**Figure 13:**
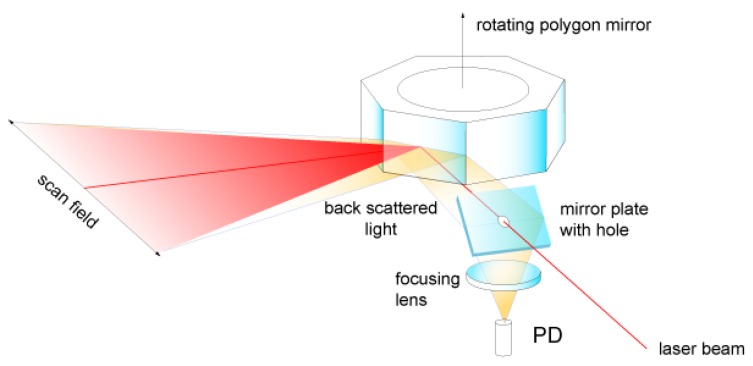
Rotating polygon mirror scanner [99].

Rotating polygon mirrors are the most simple and compact systems. They usually consist of a laser line source targeted at a 3D rotating polygon, whose faces are very reflective. Another possible configuration is using a laser point source and a rotating mirror that can also be tilted on another axis (see Figure 13). This way, other light patterns other than straight lines can be projected.

In any case, the FoV is inversely proportional to the number of faces of the polygon: the more faces, the smaller the scan angle. The deflection angle can be of up to 120°. Because of this multi-mirror characteristic, the polygon does not need to return to its initial position in order to start a new scan, it simply keeps rotating and the following face is used. Therefore, the polygon does not need to change the direction of rotation, which allows for very high rotating speeds and very short idle times. However, they are rather bulky and only allow raster scanning. Polygon mirrors have been rarely used in underwater 3D scanners [82].

#### 4.2.2. Risley or Wedge Prisms

**Figure 14:**
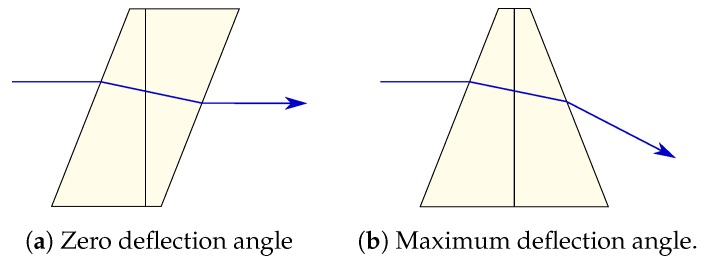
Risley prisms.

Risley prisms deflect the light beam by rotating one wedge prism with respect to the other, as shown in Figure 14. Light passes through both prisms, experimenting with a double refraction process. When the prisms are rotated such that the normal of their refractive faces are parallel, the direction of the outgoing light beam is the same as the incoming (Figure 14a). However, when one of them is rotated and their normals are not parallel anymore, the outgoing light beam is deflected (Figure 14b).

This mechanism results in a very compact and robust scanners and achieves deflection angle of up to 120°. However, their main disadvantage is that they need large rotations to obtain a significant beam deflection, especially when scanning objects close to the optical axis [100].

#### 4.2.3. Galvanometer

Galvanometer scanners use a small electromechanical element attached to a flat mirror that can quickly turn it in small-angle increments [101]. There is an inherent trade-off between the mirror size and the maximum angular velocity, which means that there is a trade-off between the laser beam size and the scanning speed [79]. Usually, they achieve deflection angles of around 80°, and their scanning frequency is below 50 Hz. Galvanometers have been used in underwater 3D scanners [48,92,102,103].

#### 4.2.4. MEMS Micromirrors

**Figure 15:**
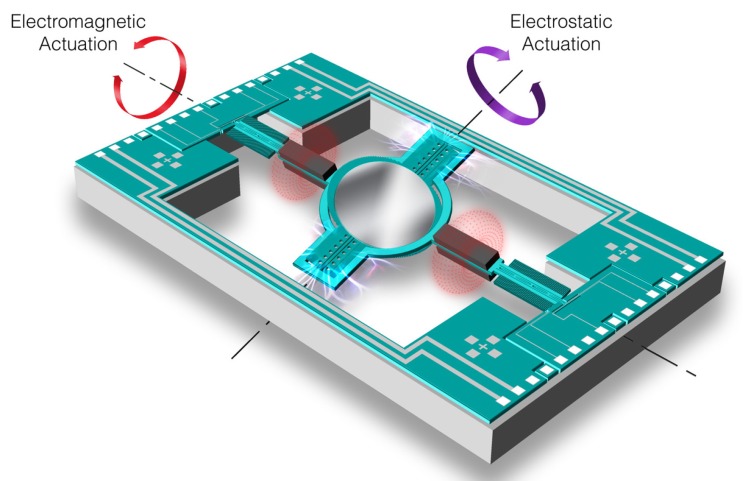
Maradin’s MEMS 2D laser scanning mirror [114].

microelectromechanical systems (MEMS) micromirrors are very similar to galvanometers, but at a smaller scale. They can work on two regimes: linear and resonant. In linear operation, they typically achieve frequencies of around 1 kHz and deflection angles of up to 30°. In resonant mode, however, they can work at higher frequencies and deflection angles by exciting one of the mirror’s axis at its resonance frequency. Nonetheless, this resonant mode is raster scanning, which means that the scene is scanned from beginning to end and arbitrary directions cannot be projected. Consequently, they cannot dynamically modify the scanned FoV in order to increase spatial resolution or decrease acquisition time. Due to their small inertia, MEMS consume less power and perform better at high resonant frequency than polygon or galvanometric scanners [104]. For more exhaustive studies on MEMS micromirrors, the reader is referred to [104,105,106]. MEMS micromirrors can be assembled in a number of configurations:

- Single biaxial MEMS scanner (also called 2D or flying spot). It consists of a single mirror that can be tilted around two axes (see Figure 15). The eigenfrequencies of the two axes are different so that they can perform resonant raster scanning at one of the natural frequencies.
- 1-dimensional array of MEMS micromirrors. It consists of several uniaxial or biaxial MEMS micromirrors, such as the one developed by Preciseley [107]. Another type of 1D array is the grating light valve (GLV)™ of Silicon Light Machines™ [108]. They act as spatial light modulators (SLMs), controlling the amount of light projected at each location of a light line. They are mostly used for displays and projectors [109].
- 2-dimensional matrix of MEMS micromirrors. They are called digital micro-mirror devices (DMDs) and are normally used as SLMs in projectors. The resolution of their projection is equal to the number of micromirrors. Each of the mirrors is bistable, so they are always either *on* or *off*. However, they can achieve shades of gray by being *on* only a fraction of the total projection time of each frame. The best known commercial product is Texas Instruments’ digital light processor (DLP) [110]. There are underwater 3D scanners that use DMDs to project patterns which are more complex than a line [111,112,113].

### 4.3. Non-Mechanical Beam Steering (Solid-State)

Mechanical beam scanners are robust and reliable. However, non-mechanical beam deflectors, also called solid-state deflectors, are being increasingly used in 3D scanners because of a number of reasons. Mainly, their mass-free nature allows them to achieve much higher deflection velocities. Moreover, they do not experience mechanical issues such as wear and drift. Although their maximum deflection angles are limited to a few degrees, they have high angular accuracy. Another positive characteristic is that they allow random-access scanning. For a more detailed study on electro–optic (EO) and acousto–optic (AO) laser beam scanners, the reader is referred to [115].

#### 4.3.1. Electro–Optic Reflector (EOD)

**Figure 16:**
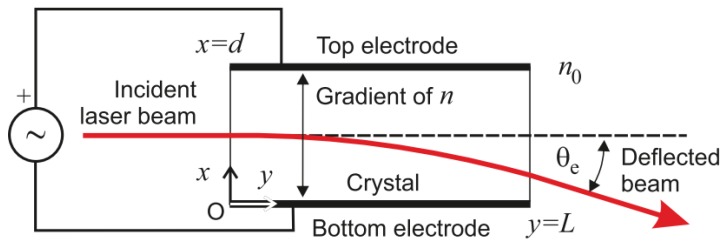
Electro–optic deflector (eod) scheme [115].

**Figure 17:**
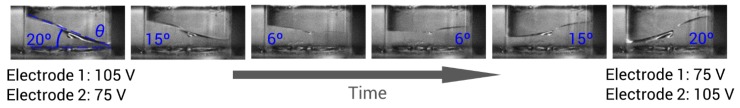
Electrowetting [120].

electro–optic deflectors (EODs) perform beam steering by changing the refractive index of an optically transparent material as a result of an electric field [116] (see Figure 16). There are different types of eo technologies:

- Liquid crystal waveguides accomplish in-plane beam steering by changing the voltage on one or more prisms filled with liquid crystals. The in-plane deflection angle can be of 60°, while out-of-plane steering is of around 15°. Their response time is of less than 500 μs. However, their main limitation is the size of the aperture of less than 1 cm [117].
- Electro-wetting-based systems use sealed cavities filled with two immiscible liquids, such as water and oil [118]. When a voltage difference is applied, the contact angle between the liquids is modified (see Figure 17), which deflects the laser beam. For large angles, light transmittance can drop to 30% [119]. Due to its high inertia, its maximum frequency of scene acquisition in a working scanner is around 2 Hz [120].
- potassium tantalate niobate (KTN) crystal has the maximum eo effects among existing materials. These devices are capable of very high-speed deflection (around 80 ns), but the maximum deflection angle is only of ±7° for ir wavelengths and only of ±1° for the visible spectrum [121]. Although only one-dimensional beam deflection has been achieved on a single ktn crystal, a 2D beam deflection can be obtained by lining up two deflectors appropriately. Nonetheless, this configurations is more complex and power-consuming.

#### 4.3.2. Acousto–Optic Deflector (AOD)

**Figure 18:**
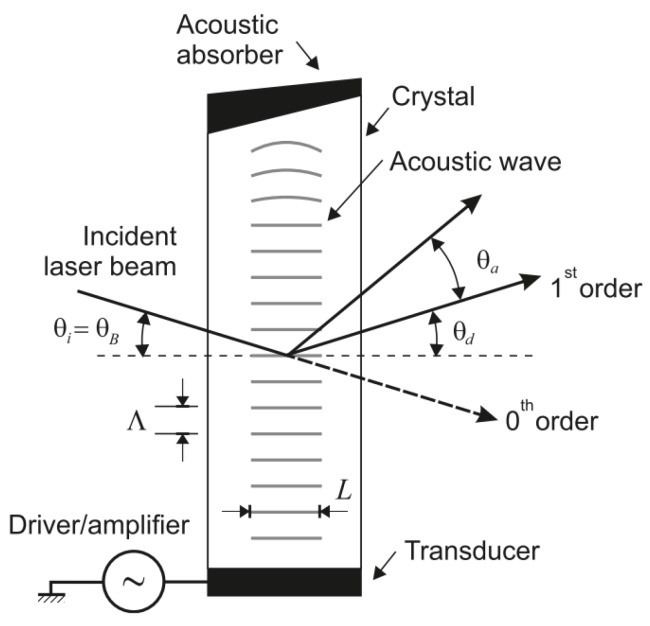
AOD scheme [115].

acousto–optic deflectors (AODs) are similar to EODs. However, they achieve the change of the refractive index of the material by propagating sound waves that induce a change of its density (see Figure 18). Their maximum deflection angle is of approximately ±2° for the visible spectrum but they are slower than ktn deflectors (access time of around 10 μs) [122]. These systems are commonly used in microscopy [123] and micromachining [124], but their small deflection angles reduce their applicability as 3D scanners for UUVs.

#### 4.3.3. Optical Phased Array

**Figure 19:**
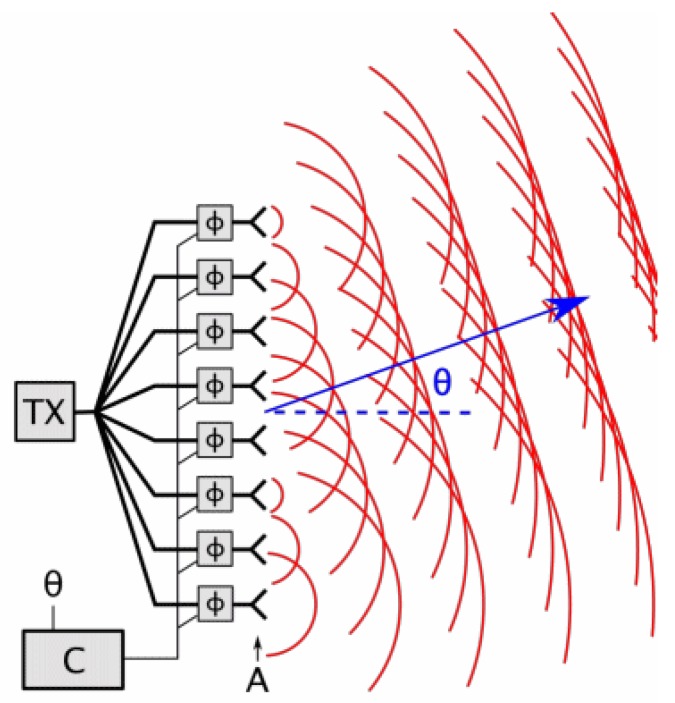
OPA scheme [129].

An optical phased array (OPA) is an array of coherent optical sources, which means that they emit signals with a constant phase difference and the same frequency and waveform [41]. The deflection angle of the beam can be controlled by the phase and/or amplitude of the emitters [125] (see Figure 19). OPAs allow scanning frequencies of around 100 kHz. However, the insertion loss of the laser power is a drawback [126,127]. One of the few examples of opa-based 3D scanner is [128], which aims at being implemented as lidar for autonomous cars. Nonetheless, these systems have not been used in real terrestrial nor aquatic vehicles to the best of the authors’ knowledge.

### 4.4. Conclusions

**Figure 20:**
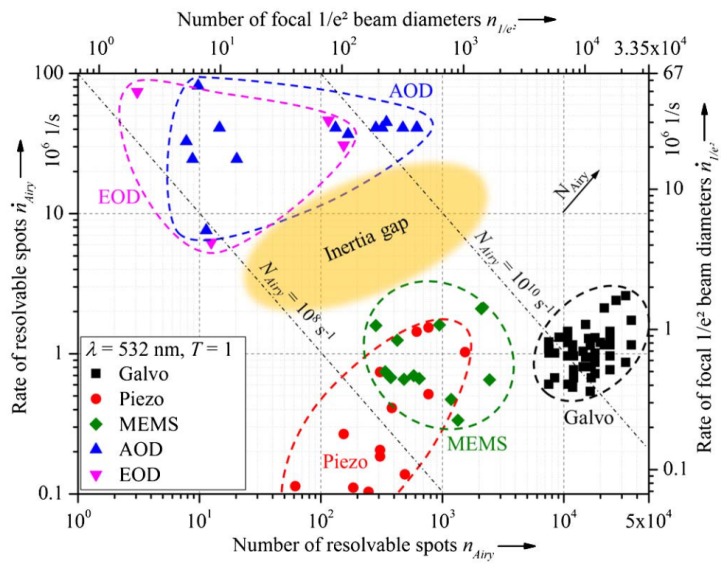
Number and rate of resolvable spots [130].

Performance comparisons of mechanical and non-mechanical beam steerers have been done by Bechtold et al. [130] and Römer and Bechtold [115]. The spatial resolutions (number of resolvable spots) versus the scanning speeds (rate of resolvable spots) of different devices are plotted in Figure 20. There is a clear division between mechanical and non-mechanical (or solid-state) laser scanners. In general, mechanical laser scanners provide higher angular resolution and maximum deflection angle, while non-mechanical devices allow for much higher speeds. In this respect, Ref. [115] proposed a combination of mechanical and non-mechanical elements could be highly useful in applications where high spatial resolution and high scanning speed are needed.

Regarding optical efficiency, mirror-based scanners have very small losses for a wide range of light wavelengths. On the other hand, EODs show better performance for a narrower range of wavelengths: for instance, ktn achieves a much higher FoV for ir light than for visible light. Furthermore, the efficiency of AODs is typically limited to around 70% [115].

In mechanical systems, there seems to be a trade-off between FoV and size. Polygon mirrors achieve a FoV of up to 120°, but they are large and heavy. Galvanometers usually provide a FoV of 80° and have a smaller size. MEMS can have a diameter of less than 1 mm, but they can typically only deflect the laser beam 30°.

Concerning systems that do not perform beam steering and scan the whole scene at once, they have the advantage of being robust against high dynamics of the scanned scene and against the sensor movement, at the cost of reducing lateral resolution. Moreover, systems that use diffuse light are limited to shorter measuring ranges due to a higher attenuation of light and an increased effect of backscatter.

## 5. Quantitative Analysis of Current Technologies

This section collects and compares active optical underwater 3D sensors present in the literature. Consequently, neither acoustic nor passive light scanners are considered for this study. Moreover, only devices developed after 2015 are taken into account since others presented before that date have already been gathered in other surveys (see Section 1).

Some systems are left out of this analysis because they follow a hybrid approach of combining active and passive light techniques. For instance, some stereo cameras make use of active light projection [113,131,132] in order to ease the feature finding and matching processes. Duda et al. [133] use an iterative combination of active light projection with sfm.

In the first subsections, the different 3D scanners are initially grouped by the type of structured light used (regardless of whether they work on ToF or triangulation principles): one-shot illumination (Section 5.1), steered line (Section 5.2), non-steered line (Section 5.3), or steered point (Section 5.4). In Section 5.5, commercial depth cameras adapted for underwater scanning are gathered. Then, a discussion comparing all the analyzed sensors is made in Section 5.6. Finally, commercial underwater solutions are collected in Section 5.7.

### 5.1. One-Shot Illumination

One-shot illumination consists of illuminating and acquiring the whole scene at once. Risholm et al. [76,134] and Chua et al. [75] used a doe to flash diffuse light in every direction with no spatial structure. Other authors employ spatially-structured light: Massot-Campos and Oliver-Codina [60] used a special doe to project 25 lines onto the surface of the scanned object, whereas Sarafraz and Haus [135] and Risholm et al. [112,136] used commercial projectors to illuminate the scene with specifically-designed, more complex patterns. Bleier and Nüchter [137] simply used two laser lines forming a cross.

The devices presented by Risholm et al. [76], Mariani et al. [134] and Chua et al. [75] are range-gated ToF sensors. This means that their light receiver opens with a delay after the light has been sent out. This is done to make them more robust against backscatter (see Figure 1 and Figure 3). Risholm et al. [76], Mariani et al. [134] developed a peak-finding algorithm that can deal with forward scatter and at the same time can perceive distant peaks that are barely higher than peaks caused by noise. This way, they can increase the theoretical depth resolution of 18.8 cm by a factor of 20. They measured the effect of scattering in a pool and the SNR versus depth resolution in sea. Chua et al. [75] introduced a new range estimation model to reduce the effects induced by distance, target reflection and range distortion based on time slicing reconstruction and bidirectional reflection distribution function (BRDF).

Maccarone et al. [138] developed a ToF scanner based on a SPADs detector array of 192×128. Both stationary and moving targets were imaged under a variety of underwater scattering conditions of up to 6.7 attenuation lengths.

Massot-Campos and Oliver-Codina [60] presented a triangulation-based laser sensor for underwater close-range 3D reconstructions that projected 25 lines simultaneously. It was tested at high turbidity conditions. In [139], they compared it with stereo vision. They found out that a stereo-based reconstruction is best suited for long, high altitude surveys, granted that the scene has enough texture and light. On the other hand, their structured light reconstruction worked better at short distances where accurate dimensions of an object or structure where needed. For testing, both sensors were mounted, one at a time, on a Cartesian robot and performed a lawn-moving survey over a pool.

Sarafraz and Haus [135] developed a triangulation-based scanner to simultaneously estimate both the geometric shape of the water surface and the geometric shape of underwater objects from outside the water. They chose a complex pattern of red, green and blue dots using a liquid crystal display (LCD) commercial projector.

Risholm et al. [112] projected a gray code phase stepping (GCPS) pattern using a LED with a DLP projector. In a newer version [136], they developed a multi-frequency phase stepping (MFPS) pattern, which was reportedly more accurate in-depth and more robust to turbidity than GCPS.

Bleier and Nüchter [137] developed a self-calibrating hand-held scanner. They projected two crossed line lasers and exploited coplanarity constraints to perform the 3D reconstruction. Their sensors use triangulation principles with a baseline between 0.5 m and 1 m. The depth accuracy of the underwater 3D reconstruction was not reported.

### 5.2. Steered Line

The scanners in this section sweep rapidly a laser line across the scene using a laser line generator and a 1-DoF galvanometer:

Chi et al. [102] first calibrated the camera’s internal parameters and the relative pose of the camera and the galvanometer in air. They also obtained several different laser plane equations to calculate the galvanometer rotating axis equation. The compensation of the double refraction was done by geometric relationships, assuming that the indices of refraction of all media were accurately known, as well as the relative distances of laser, camera, and viewport. The system was experimentally tested in a water tank by measuring spherical objects whose radii and distances had been accurately measured by a coordinate measurement machine (CMM), which represented the ground truth.

Palomer et al. [48] took into account the distortion introduced by the double refraction through flat viewports. Rather than a ray-based triangulation, they used elliptical cones to speed up the reconstruction while not increasing the error. This sensor was successfully used for manipulation [18] as well as for object recognition and SLAM tasks [16].

### 5.3. Non-Steered Line

Non-steered laser line scanners, also called profilers, always keep the same relative position of the laser plane with respect to the camera. Therefore, the whole system must be translated and/or rotated in order to scan a scene. This is usually done by attaching the sensor to a vehicle or to a robotic arm, or by moving it by hand.

Lopes et al. [140] developed a scanner with two-line lasers (red and green). The results using the red laser were reportedly more accurate than with the green one, although no explanation of the possible causes was given. Their goal was to build a general-purpose, low-cost prototype for underwater industries, but the final cost was not reported. The system was calibrated both in dry and underwater environments using two different methods, based on the cross-ratio invariance principle and on the robust fitting of the laser line projection, respectively. The second calibration method yielded more accurate results. During the tests, the scanner was not submerged, so the scan was performed from outside the water tank. In [141], the scanner was mounted on an AUV to explore underwater mines.

Constantinou et al. [142] developed a laser scanner to measure mesh-like objects, like fish nets. The system was calibrated automatically, with the help of a calibration box. Three lasers were used in order to cover a wider area. The system was mounted on a rov and tested in a pool and in real environment at an offshore aquaculture installation, where the relative position of a fishnet with respect to the rov was measured.

Matos et al. [143] assumed their laser to be perpendicular to the viewport so that the laser plane was not refracted. Moreover, the two refractions that occur between air, glass, and water for the camera were approximated as one refraction between air and water. The tests were carried out with the sensor mounted on a linear slider outside the water tank measuring two fixed spheres and a dummy head, which had been calibrated with a CMM.

Bodenmann et al. [61] developed a system to simultaneously capture both structure and color using a single camera, a line laser and diffuse led light. The line laser was fixed on an underwater vehicle pointing vertically downwards and projected a line onto the seafloor that formed a 90° angle with the forward-moving direction. Half of the camera’s FoV was devoted to laser detection and the other half to color imaging. The 3D color reconstructions of the seafloor were done with subcentimeter-order resolution at a depth of more than 2000 m. The color was added to the bathymetry by back-projecting each 3D point into the illuminated area of a photo to retrieve the RGB components for that point in space. Moreover, the texture map was corrected for the color-dependent attenuation of light in water to reproduce the colors as if the water were drained. The scanner was mainly used at a distance of 2 m, because it was found to be a good compromise between area covered and image quality: imaging at a lower altitude entailed smaller covered area while scanning at altitudes exceeding 3 m meant darker image with lower contrast. Laser detection was improved by using machine learning techniques. Then, a similar system was used to do SLAM [144].

### 5.4. Steered Point

Steered-point or flying-spot scanners are usually ToF scanners that sweep a point across the scene, building the 3D representation of the environment point by point. They typically steer the point in 2D, although they can also do it only in 1D if mounted on a moving platform.

Imaki et al. [82] chose a laser pointer and a 2-DoF polygon mirror. This approach resulted in a rather bulky system (∅20 cm × 60 cm) but achieved a wider horizontal FoV (120°). They used a pmt as a ToF receiver. They carried out performance tests in a pool and 3D reconstruction in field experiments in the ocean. In its newer version [145], the authors used a laser line generator and a 1-DoF polygon mirror, which slightly reduced size and weight while providing the same FoV and resolution. It was mounted on an AUV to map the seafloor.

Maccarone et al. [146] developed a time-gated ToF sensor that consisted on two 1D galvanometers and a single-pixel SPAD. They used the time-correlated single-photon counting (TCSPC) technique. An object in a water tank was scanned from the outside at different water turbidity levels, and scans at distances of up to eight attenuation lengths were achieved. The spatial resolution was only slightly affected as the scattering level was increased, even at eight attenuation lengths, with an angular resolution of approximately 60 μrad in water samples with a high scattering level. Regarding acquisition times, the authors only reported that they needed to be long.

### 5.5. Off-the-Shelf IR Depth Cameras

This analysis also includes three papers that proposed to use different existing ir depth cameras and make them suitable for underwater applications. They all needed to post-process the resulting data in order to correct the effect of refraction, which involved a preliminary calibration step.

Digumarti et al. [97] used Intel RealSense to develop a cheap and compact solution that enabled handheld scanning of marine life for divers. They scanned the coral reef in the ocean at a depth of 20 m. Anwer et al. [147] used Kinect v2 from above the water to measure objects in a water tank. They found out that their measurements were very noisy, so they additionally applied a median filter that could partly deal with the noise. Chourasiya et al. [148] used a Kinect v1 from outside the water to measure objects in a water tank at different turbidity levels.

### 5.6. Discussion

**Figure 21:**
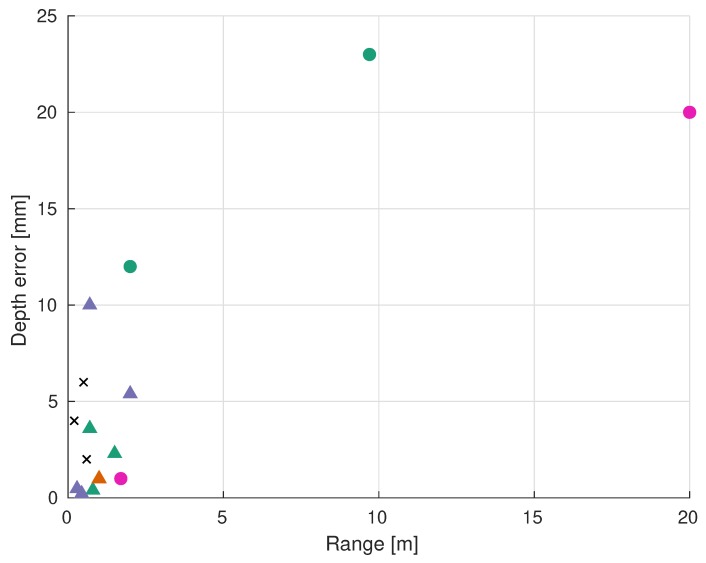
Reported absolute depth error of each system. See legend in Table 1. “X” represent off-the-shelf ir depth cameras [97,147,148].

**Figure 22:**
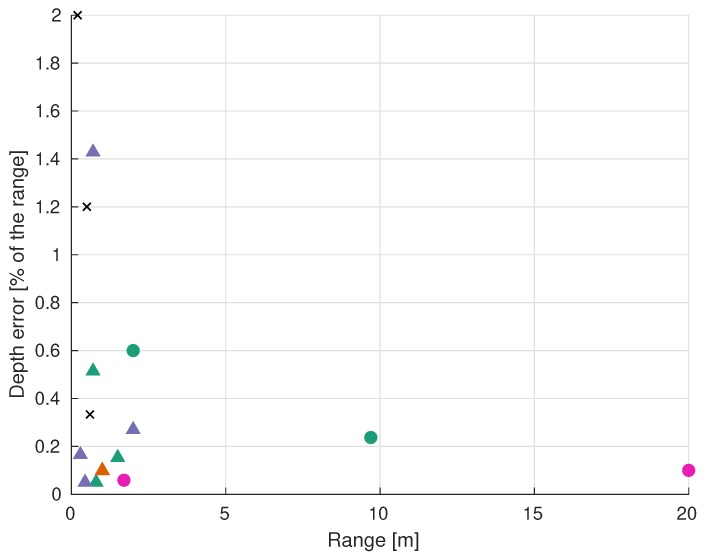
Reported relative depth error of each system. See legend in Table 1. “X” represent off-the-shelf ir depth cameras [97,147,148].

The classification of the analyzed scanners can be visualized in Table 1. Moreover, Figure 21 and Figure 22 show the absolute and relative depth error of each group of sensors. The reported performances of the scanners are gathered in Table 2.

There is a clear difference in performance between ToF and triangulation sensors. Most of the analyzed triangulation sensors achieve depth accuracies better than 5 mm, whereas the accuracies of ToF devices are in the range of a few centimeters (see Figure 21). In fact, the best absolute and relative depth accuracies are achieved by Lopes et al. [140] and Risholm et al. [136]. On the other hand, ToF sensors can work at longer ranges of up to 20 m, which makes their relative depth error at longer distances comparable to triangulation scanners working at short ranges (usually less than 2 m), as can be seen in Figure 22. Maccarone et al. [146] achieved the lowest depth error of all the ToF sensors (<1 mm), but they worked in ideal, dark conditions and with a very high acquisition time and a very small fov. The fact that the depth accuracy of triangulation sensors decreases with longer measuring ranges, unlike ToF sensors, was expected given their different working principles (see Section 3.3).

Off-the-shelf ir depth cameras are represented with “X” marks in Figure 21 and Figure 22. It can be seen that their high relative depth error and short-range make them not suitable for UUVs’ tasks such as mapping, inspection or manipulation. Nonetheless, Chourasiya et al. [148] could reportedly achieve 0.33% relative depth error under ideal conditions. In general, their low price and low development effort might make them useful in price-sensitive applications where the scanning range is a few tens of centimeters.

It must be noted that the authors of the presented papers tested depth accuracy in different ways. Some opted to directly scan a gauge object that was placed at a known distance from the sensor. Others placed the object on the floor, while the camera scanned at a known distance from the floor. Another used method consisted of scanning the same scene with another different sensor and then computing the average distance between point clouds, although in this case, the alternative method needs to have proved to be very accurate. Moreover, some authors report a relative depth accuracy of triangulation sensors with respect to the total object size, which is not as informative as with respect to the measuring range.

Regarding the used viewports, most of the analyzed sensors use flat windows. Nonetheless, some such as Imaki et al. [82] and Constantinou et al. [142] use hemispherical viewports. Flat viewports are easier to manufacture and mount, but they introduce distortions especially at high incidence angles of the laser beam in the viewport. Hemispherical or dome viewports do not in principle suffer from this distortion, but in practice, there are usually some misalignments (see Section 2.1).

The baseline of triangulation sensors is a very important design decision, as discussed in Section 3.2. The reported baselines are 100 mm (Lopes et al. [140]), 100 to 200 mm (Risholm et al. [112,136]), 265 mm (Matos et al. [143]) and 150 to 400 mm (Palomer et al. [48]). Bodenmann et al. [61] use a larger baseline between 800 and 1500 mm. The rest of authors did not inform on the baseline of their triangulation sensors.

There is a wide variety of applications to which each type of sensor can be applied. One-shot scanners acquire the whole scene at once, so there is no movement-related distortion. Therefore, they can provide undistorted data when they are mounted on moving vehicles or even moved manually. Consequently, applications include hand-held scanners for archaeological surveying or sea-life monitoring cameras. Non-steered line scanners are commonly used mounted on an UUV facing downwards to map the seafloor at a distance of a few meters. Steered line scanners are typically used to scan an object with a highly dense point cloud, which can be used in object recognition and manipulation tasks. In general, most scanners are quite versatile, so their applications are not strictly limited to the ones mentioned here.

#### 5.6.1. Other Performance Criteria

Apart from depth accuracy, there are other important performance criteria. First, the number of complete scans per second measures the refresh rate of the sensor. Scanners that are able to provide more frames per second are more suitable for scenes with faster dynamics. The reported refresh rates are 0.1 Hz (Risholm et al. [136]), 0.1 Hz to 6 Hz, depending on lateral resolution (Palomer et al. [48]), 0.2 Hz (Imaki et al. [82]), 1 Hz (Digumarti et al. [97] with Intel RealSense) and 10 Hz (Risholm et al. [76,134] and Anwer et al. [147] with Kinect v2). Typical underwater manipulation tasks require a refresh rate higher than a few hertz. Others like sealife monitoring need faster frequencies. Seafloor mapping with non-steered laser line scanners usually have refresh rates of a few tens of hertz for each line (12 Hz [61]–30 Hz [137]). Another very relevant criterion is the number of scanned points per second, which measures the trade-off between point cloud density and refresh rate. Unfortunately, many of the analyzed sensors do not report it.

Regarding the lateral resolution of ToF scanners, many authors simply report the pixel resolution of the detector. Even though it is an important parameter, the actual lateral resolution of ToF scanners is limited by the optical spreading of the beam, as depicted in Figure 1. Maccarone et al. compared a multi-pixel ToF sensor [138] with a previous single-pixel detector [146]. The multi-pixel ToF sensor provided a faster acquisition time but at the cost of a reduced spatial resolution. In the case of triangulation systems, however, subpixel laser detection algorithms can be used [149,150,151]. The lateral resolution of these devices employing subpixel methods is much more robust against the scattering effects of turbid water. In this sense, another relevant factor is the steerer’s maximum number of resolvable spots. As explained in [115], the number of resolvable spots of a beam-steering mechanism is the factor by which the maximum deflection angle exceeds the beam divergence angle. It corresponds to the number of independent spots that can be addressed across the maximum deflection angle, so it constitutes an upper limit to the lateral resolution of the scanner. It is an objective parameter to allow a comparison of the maximum deflection angles of different technologies since it is invariant with respect to imaging optics.

Another relevant parameter is the scanner’s fov. Wide FoVs are usually preferred because they can in principle provide more information about the environment. However, a larger FoV entails a longer acquisition and processing time or a reduced lateral resolution. Moreover, it also involves higher incidence angles on the viewport, which introduces error if a flat viewport is used. The FoV of laser line modules depends on the fan angle induced by the optical elements, and it can range from 30° [143] to 160° [142].

#### 5.6.2. Turbidity

Apart from the sensor’s performance, water turbidity is one of the main challenges of underwater scanning (see Section 2), and it has various effects. On the one hand, Risholm et al. [112] report a worsening in-depth precision for higher turbidity levels. On the other hand, Massot-Campos and Oliver-Codina [60] states that the effect of higher turbidity on their sensor is a major decrease in the number of detected points due to a reduction of the maximum scanning range. Consequently, only a sparse reconstruction can be performed, but they claim that this sparse reconstruction does not lose accuracy. Moreover, the performance of ToF sensors at high turbidity levels depends strongly on the reflectivity of the scanned object. SNR is much lower for non-reflective, dark surfaces, which introduces errors in the 3D reconstruction [112].

The sensor presented by Risholm et al. [76] is capable of estimating depth at distances of over 4.5 attenuation lengths when imaging high albedo (highly reflective) targets at low attenuation lengths of less than 2 m. In their case, the attenuation length of the water is measured with a specifically designed sensor. Mariani et al. [134] states that water attenuation is not the limiting factor in very clear water with attenuation lengths larger than 4.5 m. They illuminate the whole scene at once with a flash laser, whose signal primarily drops with the inverse of the distance squared due to radial spreading. Apart from attenuation lengths, turbidity is also measured in other units, such as dB lost per meter [82] or ntu. Chourasiya et al. [148] correlate maximum scan distance and turbidity: they can scan up to 10 cm at 100 ntu with Kinect^™^ v1. The data represented in Figure 21 and Figure 22 corresponds to the reported results at the lowest turbidity level of each paper (clear water).

### 5.7. Commercial Scanners

There are also commercial optical underwater 3D scanners. Their performances are compared quantitatively in Table 3. The only ToF scanners are the ones by 3DatDepth, the rest are triangulation-based. Regarding the light projection techniques, many of them (NewtonLabs, 3DatDepth, KrakenRobotics) steer the laser line. On the other hand, the scanners created by 2GRobotics and Savante perform fixed-line scanning.

Some of the performance results in Table 3 should be highlighted. Regarding range, they can all achieve longer distances than academic sensors. ToF sensors by 3DatDepth can measure at distances of up to 45 m, while 2GRobotics has develop the ULS-500 PRO [154], which allows for distances of up to 20 m. In order to achieve that, its baseline is very long (1.24 m). Most of the analyzed commercial scanners can work at water depths of a few thousand meters. Concerning depth accuracy, ToF scanners by 3DatDepth achieve errors of a few millimeters, which constitute relative errors of 0.013% at their maximum range. On the other hand, triangulation sensors, NewtonLabs’ M210UW [164] and Kraken’s SeaVision [63] achieve 0.08% and 0.15%, respectively.

## 6. Conclusions

The design of an active optical 3D sensor for underwater applications depends strongly on the characteristics of the task to be carried out. The decision of whether it should be a ToF or a triangulation scanner is mainly determined by the trade-off between depth accuracy and range: a ToF sensor is more suited for ranges up to a few tens of meters, whereas a triangulation sensor can be capable of submillimetric accuracy when working at shorter ranges.

Another design trade-off concerns lateral resolution, acquisition time and structural simplicity. One-shot systems acquire the whole scene at once—which makes them more suited for highly dynamic scenes—at the expense of reducing lateral resolution. Steered line scanners achieve very dense point clouds but if they are attached to an UUV, the movement of the vehicle can distort the resulting data. Non-steered line sensors are easier to build and calibrate, but they need to be attached to a moving platform, such as a vehicle or a rotating tripod. The accuracy of the measurements depends heavily on the accuracy of the data of the platform’s position.

It is relevant to note that some of the reviewed papers lack a complete, systematic test on the performance of their presented sensors. Therefore, quantitative comparisons of the current technologies are cumbersome to carry out. It would be highly beneficial that researchers reported on basic performance criteria of underwater 3D scanners, such as depth resolution, measurement range, lateral resolution, number of scans per second and FoV.

As explained in Section 5, depth accuracy can be assessed by statistically analyzing the data resulting from measuring known objects at known distances. For triangulation sensors, the baseline and the distance to the object at which the depth measurements were made should always be reported, since their depth resolution depends on their baseline and range. A way to experimentally assess lateral resolution of ToF scanners is as performed in [138]. The authors scanned an object that was composed of bars whose widths decreased down to zero. Then, they estimated the lateral resolution of the sensor to be equal to the smallest bar width that the system is able to resolve. In order to assess 3D scanners in a complete, objective way, authors should ideally conform to metrological standards, such as VDI/VDE 2634 [170].

Furthermore, it would be interesting to systematically test the sensors with different targets and at varying turbidity conditions, such as [60,112,134], among others, because robustness against changing visibility situations is fundamental for autonomous robots to work in real environments.

## Figures and Tables

**Table 1:**
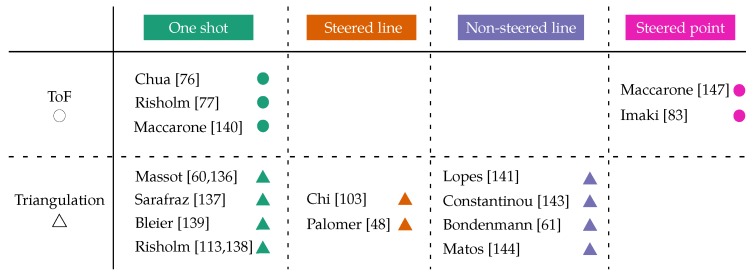
Classification of the analyzed systems.

**Table 2:** Quantitative comparison of current underwater active optical 3D scanners. For an explanation of the symbol code, please see Table 1.

Reference	Type	Depth Accuracy [mm]	Range (min–max) [m]	Depth Accuracy [% of Range]	Frequency [Hz]	FoV [°]	Baseline (min–max) [mm]
Chua, 2017 [75]	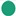	12	2	0.60	-	-	-
Risholm, 2018 [76]	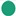	23	9.7	0.24	10	-	-
Maccarone, 2019 [138]	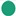	-	1.7	-	10	≈1.7×1.7	-
Massot, 2015 [60,139]	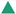	3.6	0.7	0.51	15	19	-
Sarafraz, 2016 [135]	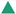	2.3	1.5	0.15	-	-	-
Bleier, 2017 [137]	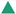	-	-	-	30/line	64	-
Risholm, 2018 [112,136]	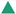	0.4	0.8 (0.5–2)	0.05	8	-	150
Chi, 2016 [102]	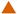	1	1 (0.7–1)	0.10	-	-	-
Palomer, 2017 [48]	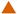	0.98	1 (0.5 – 1.2)	0.098	0.1–6	-	300 (150–400)
Lopes, 2015 [140]	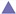	0.22	0.44 (0.2–1.5)	0.05	-	-	100
Constantinou, 2016 [142]	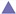	10	0.7 (0.1–1)	1.43	25/line	160	-
Bodenmann, 2017 [61]	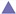	5.4	2 (0.8–3)	0.27	12/line	64	800 (800–1500)
Matos, 2019 [143]	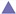	0.48	0.29 (0.15–0.29)	0.17	-	30	265
Maccarone, 2015 [146]	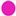	<1	1.7	0.059	-	1.6×1.6	-
Imaki, 2016 [82]	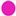	20	20	0.10	0.2	120×30	-
Digumarti, 2016 [97]	X	4	0.2	2.0	-	≈80×50	-
Anwer, 2017 [147]	X	6	0.5 (0.35–0.65)	1.20	10	-	-
Chourasiya, 2017 [148]	X	2	0.6 (0.6–1)	0.33	30	-	75

**Table 3:** Quantitative comparison of commercial underwater active optical 3D scanners. ${}^{1}$ Does not do beam steering. ${}^{2}$ After cad processing.

Company	Product	Range [m]	FoV []	Water Depth [m]	Depth Accuracy [mm]	Sample Rate [points/s]	Frequency [Hz]
2G Robotics	ULS-100 [152]	0.13–1	50 ^1^	-	-	4750	-
	ULS-200 [153]	0.36–2.5	50 ^1^	-	-	4750	-
	ULS-500 PRO [154]	1.5–20	50 ^1^	4000	-	61,440	-
	M500UW [155]	0.15–0.9	≈25 × 25	500	0.15 at 0.9 m ^2^	-	-
	HRM1500UW [156]	0.15–0.9	≈25 × 25	1500	0.15 at 0.9 m ^2^	-	-
	HRM3200UW [157]	0.15–1	≈25 × 25	3200	0.15 at 0.9 m ^2^	-	-
	HRM4000UW [158]	0.15–1	≈25 × 25	4000	0.15 at 0.9 m ^2^	-	-
	M1500UW [159]	0.5–5	≈20 × 20	1500	1.41 at 5 m ^2^	-	-
Newton Labs	M3200UW [160]	0.5–5	≈20 × 20	3200	1.41 at 5 m ^2^	-	-
	M4000UW [161]	0.5–5	≈20 x 20	4000	1.41 at 5 m ^2^	-	-
	PL3200UW-LW [162]	1.5–10	≈35 × 35	3200	1.4 at 10 m ^2^	-	-
	PL4000UW [163]	1.5–10	≈35 × 35	4000	1.4 at 10 m ^2^	-	-
	M210UW [164]	0.15–0.9	≈25 × 25	100	0.7 at 0.9 m	-	-
	M310UW [165]	0.5–5	≈20 × 20	100	1.2 at 5 m ^2^	-	-
Savante	SLV-8000i [166]	0.75–15	60 ^1^	4000	-	-	22
Kraken Robotics	SeaVision [63]	0.5–8	65 × 50	-	3 at 2 m	300 k	0.1–1
	SL1 [167]	2–45	30 × 30	3000	6	-	-
3D at Depth	SL2 [168]	2–45	30 × 30	1500	6	-	-
	SL3 [169]	2–45	30 × 30	3000	6	-	-

## Abbreviations

The following abbreviations are used in this manuscript:

ADC	analog–digital converter
AO	acousto–optic
AOD	acousto–optic deflector
APD	avalanche photodiode
AUV	autonomous underwater vehicle
BRDF	bidirectional reflection distribution function
CAD	computer-aided design
CCD	charge-coupled device
CMM	coordinate measurement machine
CMOS	complementary metal-oxide-semiconductor
CW	continuous wave
DLP	digital light processor
DMD	digital micro-mirror device
DOE	diffractive optical element
DoF	degree of freedom
EO	electro–optic
EOD	electro–optic deflector
FoV	field of view
GCPS	gray code phase stepping
GLV	grating light valve
IMR	inspection, maintenance and repair
IMU	inertial measurement unit
INS	inertial navigation system
IR	infrared
KTN	potassium tantalate niobate
LCD	liquid crystal display
LED	light emitting diode
LiDAR	light detection and ranging
LLS	laser line scanner
MEMS	microelectromechanical systems
MFPS	multi-frequency phase stepping
MPPC	multipixel photon counter
NTU	nephelometric turbidity units
OPA	optical phased array
PMT	photomultiplier tube
ROV	remotely operated vehicle
SfM	structure from motion
SiPM	silicon photomultiplier
SLAM	simultaneous localization and mapping
SLM	spatial light modulator
SNR	signal-to-noise ratio
SONAR	sound navigation ranging
SPAD	single-photon avalanche photodiode
SVP	single view-point
TCSPC	time-correlated single-photon counting
ToF	time of flight
UUV	unmanned underwater vehicle

## References

[1] National Oceanic and Atmospheric Administration (NOAA), US Depatment of Commerce Oceans & Coasts. https://www.noaa.gov/oceans-coasts.

[2] Kyo M., Hiyazaki E., Tsukioka S., Ochi H., Amitani Y., Tsuchiya T., Aoki T., Takagawa S. The sea trial of “KAIKO”, the full ocean depth research ROV. Proceedings of the OCEANS ’95 MTS/IEEE ’Challenges of Our Changing Global Environment’.

[3] Foley B., Mindell D. (2002). Precision Survey and Archaeological Methodology in Deep Water. ENALIA J. Hell. Inst. Mar. Archaeol..

[4] García R., Gracias N., Nicosevici T., Prados R., Hurtós N., Campos R., Escartin J., Elibol A., Hegedus R., Neumann L. (2017). Exploring the Seafloor with Underwater Robots. Computer Vision in Vehicle Technology.

[5] Roman C., Inglis G., Rutter J. Application of structured light imaging for high resolution mapping of underwater archaeological sites. Proceedings of the OCEANS’10 IEEE Sydney.

[6] Johnson-Roberson M., Bryson M., Friedman A., Pizarro O., Troni G., Ozog P., Henderson J.C. (2017). High-Resolution Underwater Robotic Vision-Based Mapping and Three-Dimensional Reconstruction for Archaeology. J. Field Robot..

[7] Giguere P., Dudek G., Prahacs C., Plamondon N., Turgeon K. Unsupervised learning of terrain appearance for automated coral reef exploration. Proceedings of the 2009 Canadian Conference on Computer and Robot Vision (CRV).

[8] Smith R.N., Schwager M., Smith S.L., Jones B.H., Rus D., Sukhatme G.S. (2011). Persistent ocean monitoring with underwater gliders: Adapting sampling resolution. J. Field Robot..

[9] Pizarro O., Singh H. (2003). Toward large-area mosaicing for underwater scientific applications. IEEE J. Ocean. Eng..

[10] Pascoal A., Oliveira P., Silvestre C., Sebastião L., Rufino M., Barroso V., Gomes J., Ayela G., Coince P., Cardew M. (2000). Robotic ocean vehicles for marine science applications: The european ASIMOV project. IEEE Ocean. Conf. Rec..

[11] Yoerger D.R., Jakuba M., Bradley A.M., Bingham B. (2007). Techniques for deep sea near bottom survey using an autonomous underwater vehicle. Int. J. Robot. Res..

[12] DeVault J.E. (2000). Robotic system for underwater inspection of bridge piers. IEEE Instrum. Meas. Mag..

[13] Lirman D., Gracias N., Gintert B., Gleason A.C., Deangelo G., Dick M., Martinez E., Reid R.P. (2010). Damage and recovery assessment of vessel grounding injuries on coral reef habitats by use of georeferenced landscape video mosaics. Limnol. Oceanogr. Methods.

[14] Schjølberg I., Gjersvik T.B., Transeth A.A., Utne I.B. (2016). Next Generation Subsea Inspection, Maintenance and Repair Operations. IFAC-PapersOnLine.

[15] Liljebäck P., Mills R. Eelume: A flexible and subsea resident IMR vehicle. Proceedings of the IEEE OCEANS 2017—Aberdeen.

[16] Himri K., Ridao P., Gracias N., Palomer A., Palomeras N., Pi R. (2018). Semantic SLAM for an AUV using object recognition from point clouds. IFAC-PapersOnLine.

[17] Palomer A., Ridao P., Ribas D. (2019). Inspection of an Underwater Structure using Point Cloud SLAM with an AUV and a Laser Scanner. J. Field Robot..

[18] Palomer A., Ridao P., Youakim D., Ribas D., Forest J., Petillot Y., Peñalver Monfort A., Sanz P.J. (2018). 3D Laser Scanner for Underwater Manipulation. Sensors.

[19] Dalgleish F.R., Tetlow S., Allwood R.L. (2004). Experiments in laser-assisted visual sensing for AUV navigation. Control Eng. Pract..

[20] Massot-Campos M., Oliver-Codina G. (2015). Optical sensors and methods for underwater 3D reconstruction. Sensors.

[21] Bruno F., Bianco G., Muzzupappa M., Barone S., Razionale A.V. (2011). Experimentation of structured light and stereo vision for underwater 3D reconstruction. ISPRS J. Photogramm. Remote Sens..

[22] Bianco G., Gallo A., Bruno F., Muzzupappa M. (2013). A comparative analysis between active and passive techniques for underwater 3D reconstruction of close-range objects. Sensors.

[23] Menna F., Agrafiotis P., Georgopoulos A. (2018). State of the art and applications in archaeological underwater 3D recording and mapping. J. Cult. Herit..

[24] Seitz S. (1999). An Overview of Passive Vision Techniques.

[25] Sarafraz A., Negahdaripour S., Schechner Y.Y. (2010). Improving Stereo Correspondence in Scattering Media by Incorporating Backscatter Cue.

[26] Murez Z., Treibitz T., Ramamoorthi R., Kriegman D.J. (2015). Photometric Stereo in a Scattering Medium. IEEE Trans. Pattern Anal. Mach. Intell..

[27] Fujimura Y., Iiyama M., Hashimoto A., Minoh M. Photometric Stereo in Participating Media Considering Shape-Dependent Forward Scatter. Proceedings of the IEEE Conference on Computer Vision and Pattern Recognition.

[28] Dalgleish F.R., Caimi F.M., Britton W.B., Andren C.F. (2009). Improved LLS imaging performance in scattering-dominant waters. Ocean Sens. Monit..

[29] Johnson-Roberson M., Pizarro O., Williams S.B., Mahon I. (2010). Generation and visualization of large-scale three-dimensional reconstructions from underwater robotic surveys. J. Field Robot..

[30] Menna F., Nocerino E., Troisi S., Remondino F. (2013). A photogrammetric approach to survey floating and semi-submerged objects. Videomet. Range Imaging Appl. XII Automat. Visual Inspection.

[31] Yamafune K., Torres R., Castro F. (2017). Multi-Image Photogrammetry to Record and Reconstruct Underwater Shipwreck Sites. J. Archaeol. Method Theory.

[32] Bosch J., Istenic K., Gracias N., Garcia R., Ridao P. (2019). Omnidirectional Multicamera Video Stitching Using Depth Maps. IEEE J. Ocean. Eng..

[33] Jaffe J.S., Moore K.D., McLean J.W., Strand M. (2001). Underwater Optical Imaging: Status and Prospects. Oceanography.

[34] Kocak D.M., Caimi F.M. (2005). The Current Art of Underwater Imaging- With a Glimpse of the Past and Vision of the Future. Mar. Technol. Soc. J..

[35] Caimi F.M., Kocak D.M., Dalgleish F.R., Watson J. Underwater imaging and optics: Recent advances. Proceedings of the OCEANS 2008.

[36] Hale G.M., Querry M.R. (1973). Optical Constants of Water in the 200-nm to 200-μm Wavelength Region. Appl. Opt..

[37] Smith R.C., Baker K.S. (1981). Optical properties of the clearest natural waters (200–800 nm). Appl. Opt..

[38] Gracias N., Negahdaripour S., Neumann L., Prados R., Garcia R. A motion compensated filtering approach to remove sunlight flicker in shallow water images. Proceedings of the OCEANS 2008.

[39] Cho M., Javidi B. (2010). Three-Dimensional Visualization of Objects in Turbid Water Using Integral Imaging. J. Disp. Technol..

[40] Wikimedia Commons (2016). File: Absorption Spectrum of Liquid Water.png—Wikimedia Commons, the Free Media Repository. https://commons.wikimedia.org/w/index.php?title=File:Absorption_spectrum_of_liquid_water.png&oldid=183806579.

[41] Hecht E. (2017). Optics.

[42] Wilde F., Radtke D., Gibs J., Iwatsubo R. (1998). National Field Manual for the Collection of Water-Quality Data.

[43] Tanabashi M., Hagiwara K., Hikasa K. (2018). Review of Particle Physics. Phys. Rev. D.

[44] Kocak D.M., Dalgleish F.R., Caimi F.M., Schechner Y.Y. (2008). A Focus on Recent Developments and Trends in Underwater Imaging. Mar. Technol. Soc. J..

[45] Tan C.S., Seet G.G., Sluzek A., He D.M. (2005). A novel application of range-gated underwater laser imaging system (ULIS) in near-target turbid medium. Opt. Lasers Eng..

[46] Schechner Y.Y., Karpel N. Clear underwater vision. Proceedings of the 2004 IEEE Computer Society Conference on Computer Vision and Pattern Recognition.

[47] Hildebrandt M., Kerdels J., Albiez J., Kirchner F. A practical underwater 3D-Laserscanner. Proceedings of the OCEANS.

[48] Palomer A., Ridao P., Forest J., Ribas D. (2019). Underwater Laser Scanner: Ray-based Model and Calibration. IEEE/ASME Trans. Mechatronics.

[49] Brown D.C. (1971). Close-range camera calibration. Photogramm. Eng..

[50] Shortis M.R. (2015). Calibration techniques for accurate measurements by underwater camera systems. Sensors.

[51] Sedlazeck A., Koch R. (2012). Perspective and Non-Perspective Camera Models in Underwater Imaging—Overview and Error Analysis.

[52] Schiebener P., Straub J., Levelt Sengers J., Gallagher J. (1990). Refractive index of water and steam as function of wavelength, temperature and density. J. Phys. Chem. Ref. Data.

[53] Tengesdal O.A. (2012). Measurement of Seawater Refractive Index and Salinity by Means of Optical Refraction. Ph.D. Thesis.

[54] Łuczyński T., Pfingsthorn M., Birk A. (2017). The Pinax-model for accurate and efficient refraction correction of underwater cameras in flat-pane housings. Ocean Eng..

[55] Kwon Y.H. (1999). Object plane deformation due to refraction in two-dimensional underwater motion analysis. J. Appl. Biomech..

[56] Wang C.C., Cheng M.S. (2007). Nonmetric camera calibration for underwater laser scanning system. IEEE J. Ocean. Eng..

[57] Palomer A., Ridao P., Ribas D., Forest J. (2017). Underwater 3D Laser Scanners: The Deformation of the Plane.

[58] Kunz C., Singh H. Hemispherical refraction and camera calibration in underwater vision. Proceedings of the OCEANS 2008.

[59] Menna F., Nocerino E., Remondino F. (2017). Flat versus hemispherical dome ports in underwater photogrammetry. Int. Arch. Photogramm. Remote Sens. Spat. Inf. Sci. ISPRS Arch..

[60] Massot-Campos M., Oliver-Codina G. Underwater laser-based structured light system for one-shot 3D reconstruction. Proceedings of the IEEE Sensors.

[61] Bodenmann A., Thornton B., Ura T. (2017). Generation of High-resolution Three-dimensional Reconstructions of the Seafloor in Color using a Single Camera and Structured Light. J. Field Robot..

[62] Yang Y., Zheng B., Kan L.Y., Yu J., Wang J.C. (2014). 3D color reconstruction based on underwater RGB laser line scanning system. Optik.

[63] Kraken Robotics SeaVision®. https://krakenrobotics.com/products/seavision/.

[64] Bryson M., Johnson-Roberson M., Pizarro O., Williams S.B. (2016). True Color Correction of Autonomous Underwater Vehicle Imagery. J. Field Robot..

[65] Peng Y.T., Cosman P.C. (2017). Underwater Image Restoration Based on Image Blurriness and Light Absorption. IEEE Trans. Image Process..

[66] Lu H., Li Y., Zhang Y., Kim H., Serikawa S., Chen M. (2017). Underwater Optical Image Processing: A Comprehensive Review. Mob. Netw. Appl..

[67] Ancuti C.O., Ancuti C., De Vleeschouwer C., Neumann L., Garcia R. Color transfer for underwater dehazing and depth estimation. Proceedings of the International Conference on Image Processing.

[68] Liu J.J., Jakas A., Al-Obaidi A., Liu Y. Practical issues and development of underwater 3D laser scanners. Proceedings of the 15th IEEE International Conference on Emerging Technologies and Factory Automation (ETFA 2010).

[69] Forest J., Salvi J., Cabruja E., Pous C. Laser stripe peak detector for 3D scanners. A FIR filter approach. Proceedings of the International Conference on Pattern Recognition.

[70] Duda A., Albiez J. Back Projection Algorithm for Line Structured Light Extraction. Proceedings of the 2013 OCEANS.

[71] Zanuttigh P., Mutto C.D., Minto L., Marin G., Dominio F., Cortelazzo G.M. (2016). Time-of-Flight and Structured Light Depth Cameras: Technology and Applications.

[72] Lange R. (2000). 3D Time-of-Flight Distance Measurement with Custom Solid-State Image Sensors in CMOS/CCD-Technology. Ph.D. Thesis.

[73] Hansard M., Lee S., Choi O., Horaud R. (2012). Time of Flight Cameras: Principles, Methods, and Applications.

[74] Giancola S., Valenti M., Sala R. (2018). A Survey on 3D Cameras: Metrological Comparison of Time-of-Flight, Structured-Light and Active Stereoscopy Technologies.

[75] Chua S.Y., Guo N., Tan C.S., Wang X. (2017). Improved range estimation model for three-dimensional (3D) range gated reconstruction. Sensors.

[76] Risholm P., Thorstensen J., Thielemann J.T., Kaspersen K., Tschudi J., Yates C., Softley C., Abrosimov I., Alexander J., Haugholt K.H. (2018). Real-time super-resolved 3D in turbid water using a fast range-gated CMOS camera. Appl. Opt..

[77] Li K.K., Law H., Soileau M.J. (2012). Picosecond Ingaas PIN Photodiode For 0.95 um–1.65 um Operation. Ultrashort Pulse Spectroscopy and Applications.

[78] Kharraz O., Forsyth D. (2013). Performance comparisons between PIN and APD photodetectors for use in optical communication systems. Optik.

[79] Riu J. (2018). Cámara LiDAR de Escaneo MEMS para Imagen 3D de Resolución Espacial Variable. Ph.D. Thesis.

[80] Rumbaugh L.K., Bollt E.M., Jemison W.D., Li Y. A 532 nm Chaotic Lidar Transmitter for High Resolution Underwater Ranging and Imaging. Proceedings of the 2013 OCEANS.

[81] Dalgleish F.R., Caimi F.M., Britton W.B., Andren C.F. An AUV-deployable pulsed laser line scan (PLLS) imaging sensor. Proceedings of the Oceans Conference Record.

[82] Imaki M., Ochimizu H., Tsuji H., Kameyama S., Saito T., Ishibashi S., Yoshida H. (2016). Underwater three-dimensional imaging laser sensor with 120-deg wide-scanning angle using the combination of a dome lens and coaxial optics. Opt. Eng..

[83] Finkelstein H., Hsu M.J., Esener S. (2006). An ultrafast Geiger-mode single-photon avalanche diode in 0.18-μm CMOS technology. Adv. Photon Count. Tech..

[84] Yamamoto K., Yamamura K., Sato K., Kamakura S., Ota T., Suzuki H., Ohsuka S. Development of Multi-Pixel Photon Counter (MPPC). Proceedings of the IEEE Nuclear Science Symposium Conference Record.

[85] Nassalski A., Moszyński M., Syntfeld-Kazuch A., Szcześniak T., Świderski L., Wolski D., Batsch T., Baszak J. (2010). Multi pixel photon counters (MPPC) as an alternative to APD in PET applications. IEEE Trans. Nucl. Sci..

[86] Agishev R., Comerón A., Bach J., Rodriguez A., Sicard M., Riu J., Royo S. (2013). Lidar with SiPM: Some capabilities and limitations in real environment. Opt. Laser Technol..

[87] Kim H., Handa A., Benosman R., Ieng S.H., Davison A., Valstar M., French A., Pridmore T. (2014). Simultaneous Mosaicing and Tracking with an Event Camera. Proceedings of the British Machine Vision Conference.

[88] Bardow P., Davison A.J., Leutenegger S. Simultaneous Optical Flow and Intensity Estimation from an Event Camera. Proceedings of the 2016 IEEE Conference on Computer Vision and Pattern Recognition (CVPR).

[89] Kim H., Leutenegger S., Davison A.J. (2016). Real-Time 3D Reconstruction and 6-DoF Tracking With an Event Camera.

[90] Gallego G., Lund J.E., Mueggler E., Rebecq H., Delbruck T., Scaramuzza D. (2018). Event-Based, 6-DOF Camera Tracking from Photometric Depth Maps. IEEE Trans. Pattern Anal. Mach. Intell..

[91] Falanga D., Kim S., Scaramuzza D. (2019). How Fast Is Too Fast? The Role of Perception Latency in High-Speed Sense and Avoid. IEEE Robot. Autom. Lett..

[92] Moore K.D., Jaffe J.S., Ochoa B.L. (2000). Development of a new underwater bathymetric laser imaging system: L-Bath. J. Atmos. Ocean. Technol..

[93] Munaro M., So E.W.Y., Tonello S., Menegatti E. (2015). Efficient completeness inspection using real-time 3D color reconstruction with a dual-laser triangulation system. Integrated Imaging and Vision Techniques for Industrial Inspection: Advances and Applications.

[94] Ji Z., Leu M.C. (1989). Design of optical triangulation devices. Opt. Laser Technol..

[95] Narasimhan S.G., Nayar S.K. Structured light methods for underwater imaging: Light stripe scanning and photometric stereo. Proceedings of the MTS/IEEE OCEANS.

[96] McLeod D., Jacobson J., Hardy M., Embry C. Autonomous inspection using an underwater 3D LiDAR. Proceedings of the 2013 OCEANS.

[97] Digumarti S.T., Chaurasia G., Taneja A., Siegwart R., Thomas A., Beardsley P. Underwater 3D capture using a low-cost commercial depth camera. Proceedings of the 2016 IEEE Winter Conference on Applications of Computer Vision, WACV 2016.

[98] HOLO/OR LTD FOV Magnification Module. https://www.holoor.co.il/products/structured-light-module/.

[99] Dr. Walter Luhs. © LEYBOLD/LD DIDACTIC GmbH, Hürth. Photonics: Rotating Polygon Mirror Scanner. http://www.photonics.ld-didactic.com/Educational%20Kits/P5889.html.

[100] Schwarze C. (2006). A new look at Risley prisms. Photonics Spectra.

[101] Montagu J.I. (1986). Achieving optimal high resolution in galvanometric scanning systems. Infrared Technol. Appl. Int. Soc. Opt. Photonics.

[102] Chi S., Xie Z., Chen W. (2016). A Laser Line auto-scanning system for underwater 3D reconstruction. Sensors.

[103] Chantler M.J. (1997). Calibration and operation of an underwater laser triangulation sensor: The varying baseline problem. Opt. Eng..

[104] Holmström S.T., Baran U., Urey H. (2014). MEMS laser scanners: A review. J. Microelectromech. Syst..

[105] Brown M., Urey H. (2015). MEMS Microdisplays. Handbook of Visual Display Technology.

[106] Song Y., Panas R.M., Hopkins J.B. (2018). A review of micromirror arrays. Precis. Eng..

[107] Preciseley MEMS Mirror Array. https://www.preciseley.com/mems-mirror-array.html.

[108] Corrigan R., Cook R., Favotte O. (2001). Silicon Light Machines™-Grating Light Valve™ Technology Brief Breakthrough MEMS Component Technology for Optical Networks.

[109] Perry T. (2004). Tomorrow’s TV. IEEE Spectr..

[110] Hornbeck L.J. (2001). The DMD™ Projection Display Chip: A MEMS-Based Technology. MRS Bull..

[111] Narasimhan S.G., Nayar S.K., Sun B., Koppal S.J. Structured light in scattering media. Proceedings of the IEEE International Conference on Computer Vision.

[112] Risholm P., Kirkhus T., Thielemann J.T. High-resolution structured light 3D sensor for autonomous underwater inspection. Proceedings of the OCEANS 2018 MTS/IEEE.

[113] Detry R., Koch J., Pailevanian T., Garrett M., Levine D., Yahnker C., Gildner M. Turbid-water subsea infrastructure 3D reconstruction with assisted stereo. Proceedings of the 2018 OCEANS-MTS/IEEE Kobe Techno-Oceans 2018.

[114] Maradin MEMS 2D Laser Scanning Mirror. http://www.maradin.co.il/products/mar1100-mems-2d-laser-scanning-mirror/.

[115] Römer G.R., Bechtold P. (2014). Electro-optic and acousto-optic laser beam scanners. Phys. Procedia.

[116] Maldonado T.A. (1995). Electro-Optic modulators. Handb. Opt..

[117] National Research Council (2014). Laser Radar: Progress and Opportunities in Active Electro-Optical Sensing.

[118] Hou L., Smith N.R., Heikenfeld J. Electrowetting micro-prisms and micro-mirrors. Proceedings of the Lasers and Electro-Optics Society Annual Meeting-LEOS.

[119] Han W., Haus J.W., McManamon P., Heikenfeld J., Smith N., Yang J. (2010). Transmissive beam steering through electrowetting microprism arrays. Opt. Commun..

[120] Zohrabi M., Cormack R.H., Supekar O.D., Lim W.Y., Gopinath J.T., Bright V.M. (2019). Lidar system with nonmechanical electrowetting-based wide-angle beam steering. Opt. Express.

[121] Chao J.H., Zhu W., Chen C.J., Hoffman R.C., Campbell A.L., Henry M.G., Yin S. (2017). High speed non-mechanical two-dimensional KTN beam deflector enabled by space charge and temperature gradient deflection. Opt. Express.

[122] ISOMET (2018). Acousto-Optic Scanning and Deflection.

[123] Salomé R., Kremer Y., Dieudonné S., Léger J.F., Krichevsky O., Wyart C., Chatenay D., Bourdieu L. (2006). Ultrafast random-access scanning in two-photon microscopy using acousto-optic deflectors. J. Neurosci. Methods.

[124] Ngoi B.A., Venkatakrishnan K., Lim L., Tan B. (2001). Angular dispersion compensation for acousto-optic devices used for ultrashort-pulsed laser micromachining. Opt. Express.

[125] Heck M.J. (2017). Highly integrated optical phased arrays: Photonic integrated circuits for optical beam shaping and beam steering. Nanophotonics.

[126] Yaacobi A., Sun J., Moresco M., Leake G., Coolbaugh D., Watts M.R. (2014). Integrated phased array for wide-angle beam steering. Opt. Lett..

[127] Yoo H.W., Druml N., Brunner D., Schwarzl C., Thurner T., Hennecke M., Schitter G. (2018). MEMS-based lidar for autonomous driving. Elektrotechnik Und Informationstechnik.

[128] Poulton C.V., Russo P., Timurdogan E., Whitson M., Byrd M.J., Hosseini E., Moss B., Su Z., Vermeulen D., Watts M.R. High-Performance Integrated Optical Phased Arrays for Chip-Scale Beam Steering and LiDAR. Proceedings of the Conference on Lasers and Electro-Optics.

[129] Wikimedia Commons (2019). File: Phased Array Animation with Arrow 10frames 371x400px 100ms.gif—Wikimedia Commons, the Free Media Repository. https://en.wikipedia.org/wiki/File:Phased_array_animation_with_arrow_10frames_371x400px_100ms.gif.

[130] Bechtold P., Hohenstein R., Schmidt M. (2013). Evaluation of disparate laser beam deflection technologies by means of number and rate of resolvable spots. Opt. Lett..

[131] Ekkel T., Schmik J., Luhmann T., Hastedt H. (2015). Precise laser-based optical 3D measurement of welding seams under water. Int. Arch. Photogram. Remote Sens. Spat. Inf. Sci. ISPRS Arch..

[132] Buschinelli P.D., Matos G., Pinto T., Albertazzi A. Underwater 3D shape measurement using inverse triangulation through two flat refractive surfaces. Proceedings of the OCEANS 2016 MTS/IEEE Monterey.

[133] Duda A., Schwendner J., Gaudig C. SRSL: Monocular self-referenced line structured light. Proceedings of the IEEE International Conference on Intelligent Robots and Systems.

[134] Mariani P., Quincoces I., Haugholt K.H., Chardard Y., Visser A.W., Yates C., Piccinno G., Reali G., Risholm P., Thielemann J.T. (2018). Range-Gated Imaging System for Underwater Monitoring in Ocean Environment. Sustainability.

[135] Sarafraz A., Haus B.K. (2016). A structured light method for underwater surface reconstruction. ISPRS J. Photogramm. Remote Sens..

[136] Risholm P., Kirkhus T., Thielemann J.T., Thorstensen J. (2019). Adaptive Structured Light with Scatter Correction for High-Precision Underwater 3D Measurements. Sensors.

[137] Bleier M., Nüchter A. (2017). Low-Cost 3D laser scanning in air or water using self-calibrating structured light. Int. Arch. Photogram. Remote Sens. Spat. Inf. Sci. ISPRS Arch..

[138] Maccarone A., Mattioli F., Rocca D., Mccarthy A., Henderson R., Buller G.S. (2019). Three-dimensional imaging of stationary and moving targets in turbid underwater environments using a single-photon detector array. Opt. Express.

[139] Massot-Campos M., Oliver-Codina G., Kemal H., Petillot Y., Bonin-Font F. Structured light and stereo vision for underwater 3D reconstruction. Proceedings of the MTS/IEEE OCEANS 2015—Genova: Discovering Sustainable Ocean Energy for a New World.

[140] Lopes F., Silva H., Almeida J.M., Martins A., Silva E. Structured light system for underwater inspection operations. Proceedings of the MTS/IEEE OCEANS 2015—Genova: Discovering Sustainable Ocean Energy for a New World.

[141] Martins A., Almeida J., Almeida C., Dias A., Dias N., Aaltonen J., Heininen A., Koskinen K.T., Rossi C., Dominguez S. UX 1 system design—A robotic system for underwater mining exploration. Proceedings of the 2018 IEEE/RSJ International Conference on Intelligent Robots and Systems (IROS).

[142] Constantinou C.C., Loizou S.G., Georgiades G.P. An underwater laser vision system for relative 3-D posture estimation to mesh-like targets. Proceedings of the IEEE International Conference on Intelligent Robots and Systems.

[143] Matos G., Buschinelli P.D., Pinto T. (2019). Underwater Laser Triangulation Sensor Model with Flat Refractive Interfaces. IEEE J. Ocean. Eng..

[144] Massot-Campos M., Oliver G., Bodenmann A., Thornton B. Submap bathymetric SLAM using structured light in underwater environments. Proceedings of the 2016 IEEE/OES Autonomous Underwater Vehicles 2016 (AUV 2016).

[145] Ishibashi S., Ohta Y., Sugesawa M., Tanaka K., Yoshida H., Choi S. Seabed 3D images created by an underwater laser scanner applied to an AUV. Proceedings of the OCEANS 2017.

[146] Maccarone A., McCarthy A., Ren X., Warburton R.E., Wallace A.M., Moffat J., Petillot Y., Buller G.S. (2015). Underwater depth imaging using time-correlated single-photon counting. Opt. Express.

[147] Anwer A., Azhar Ali S.S., Khan A., Meriaudeau F. (2017). Underwater 3-D Scene Reconstruction Using Kinect v2 Based on Physical Models for Refraction and Time of Flight Correction. IEEE Access.

[148] Chourasiya S., Mohapatra P.K., Tripathi S. (2017). Non-intrusive underwater measurement of mobile bottom surface. Adv. Water Resour..

[149] Izquierdo M.A., Sanchez M.T., Ibañez A., Ullate L.G. (1999). Sub-pixel measurement of 3D surfaces by laser scanning. Sens. Actuators A Phys..

[150] Forest Collado J. (2005). New Methods for Triangulation-Based Shape Acquisition Using Laser Scanners. Ph.D. Thesis.

[151] de Dominicis L. (2013). Underwater 3D vision, ranging and range gating. Subsea Optics and Imaging.

[152] 2G Robotics ULS-100. https://www.2grobotics.com/products/underwater-laser-scanner-uls-100/.

[153] 2G Robotics ULS-200. https://www.2grobotics.com/products/underwater-laser-scanner-uls-200/.

[154] 2G Robotics ULS-500 PRO. https://www.2grobotics.com/products/underwater-laser-scanner-uls-500/.

[155] Newton Labs M500UW. http://www.newtonlabs.com/M500UW_landing.htm.

[156] Newton Labs HRM1500UW. http://www.newtonlabs.com/HRM1500UW_landing.htm.

[157] Newton Labs HRM3200UW. http://www.newtonlabs.com/HRM3200UW_landing.htm.

[158] Newton Labs HRM4000UW. http://www.newtonlabs.com/HRM4000UW_landing.htm.

[159] Newton Labs M1500UW. http://www.newtonlabs.com/M1500UW_landing.htm.

[160] Newton Labs M3200UW. http://www.newtonlabs.com/M3200UW_landing.htm.

[161] Newton Labs M4000UW. http://www.newtonlabs.com/M4000UW_landing.htm.

[162] Newton Labs PL3200UW-LW. http://www.newtonlabs.com/PL3200UW-LW_landing.htm.

[163] Newton Labs PL4000UW. http://www.newtonlabs.com/PL4000UW_landing.htm.

[164] Newton Labs M210UW. http://www.newtonlabs.com/scan_m200uw_sys_specs.html.

[165] Newton Labs M310UW. http://www.newtonlabs.com/scan_m300uw_sys_specs.html.

[166] Savante SLV-8000i. https://www.savante.co.uk/slv80-long-range-subsea-laser-profiler.

[167] 3D at Depth SL1 LiDAR Laser. https://www.3datdepth.com/product/sl1-lidar-laser.

[168] 3D at Depth SL2 LiDAR Laser. https://www.3datdepth.com/product/sl2-lidar-laser.

[169] 3D at Depth SL3 LiDAR Laser. https://www.3datdepth.com/product/sl3-lidar-laser.

[170] Verein Deutscher Ingenieure VDI/VDE 2634: Optical 3-D Measuring Systems. https://standards.globalspec.com/std/9914533/vdi-vde-2634-blatt-2.

